# Modelling Autism Spectrum Disorder (ASD) and Attention-Deficit/Hyperactivity Disorder (ADHD) Using Mice and Zebrafish

**DOI:** 10.3390/ijms23147550

**Published:** 2022-07-07

**Authors:** Godfried Dougnon, Hideaki Matsui

**Affiliations:** Department of Neuroscience of Disease, Brain Research Institute, Niigata University, Niigata 951-8585, Japan; dougnong@bri.niigata-u.ac.jp

**Keywords:** zebrafish, mouse, autism spectrum disorders, attention-deficit/hyperactivity disorder, neurodevelopmental disorders

## Abstract

Autism spectrum disorders (ASD) and attention-deficit/hyperactivity disorder (ADHD) are two debilitating neurodevelopmental disorders. The former is associated with social impairments whereas the latter is associated with inattentiveness, hyperactivity, and impulsivity. There is recent evidence that both disorders are somehow related and that genes may play a large role in these disorders. Despite mounting human and animal research, the neurological pathways underlying ASD and ADHD are still not well understood. Scientists investigate neurodevelopmental disorders by using animal models that have high similarities in genetics and behaviours with humans. Mice have been utilized in neuroscience research as an excellent animal model for a long time; however, the zebrafish has attracted much attention recently, with an increasingly large number of studies using this model. In this review, we first discuss ASD and ADHD aetiology from a general point of view to their characteristics and treatments. We also compare mice and zebrafish for their similarities and discuss their advantages and limitations in neuroscience. Finally, we summarize the most recent and existing research on zebrafish and mouse models of ASD and ADHD. We believe that this review will serve as a unique document providing interesting information to date about these models, thus facilitating research on ASD and ADHD.

## 1. Introduction

Autism spectrum disorders (ASD) and attention-deficit/hyperactivity disorder (ADHD) are two distinct neurodevelopmental disorders that share symptoms and genes, making it difficult to understand and separate them. Common signs and symptoms include difficulty paying attention and problems related to concentration, activity, and relationships. However, although the same individual can have both conditions, there are key differences in their prevalence, causes, diagnostics, and treatment therapies. ASD or autism refers to a broad range of conditions characterized by repetitive behaviours and difficulties in social skills, speech, and nonverbal communication. There are several subtypes of autism caused by a combination of genetic and environmental influences, whereas ADHD (also known as ADD) is not a spectrum disorder. Moreover, these disorders can cause a range of difficulties from one individual to another. To research therapies for these disorders, animal models are often used, especially mouse and zebrafish models. This review focuses on first describing the two disorders and then presenting existing models of ASD and ADHD in mice and zebrafish.

### 1.1. Overview and Epidemiology of Autism Spectrum Disorder (ASD)

#### 1.1.1. Overview

Autism or ASD is a neurological and developmental disorder affecting individuals in diverse aspects of their life, such as interaction, communication, learning and social behaviour. Symptoms are usually detected in early life (~2 years), and according to the Diagnostic and Statistical Manual of Mental Disorders (DSM-5) [1], individuals are diagnosed with ASD if they demonstrate:

- Difficulty communicating and interacting with others.
- Limited interests and repetitive behaviours.
- Troubles in functioning at school, work, and in society.

Although there is no cure for ASD to date, treatments and services can improve an individual’s symptoms and daily life.

#### 1.1.2. Prevalence

**Figure 1:**
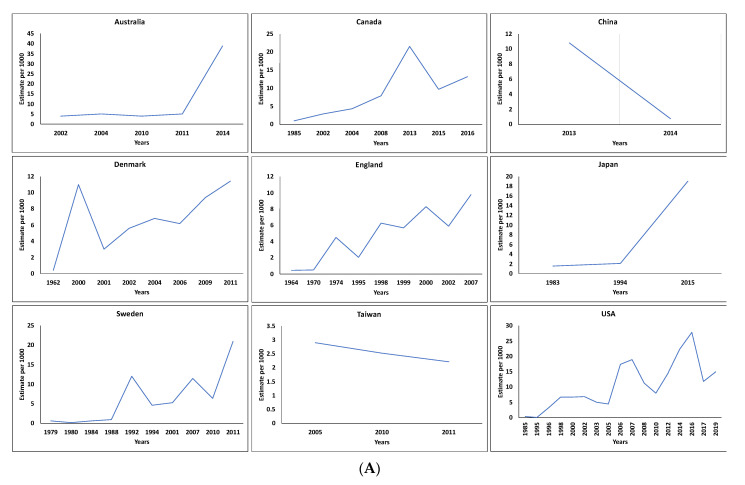

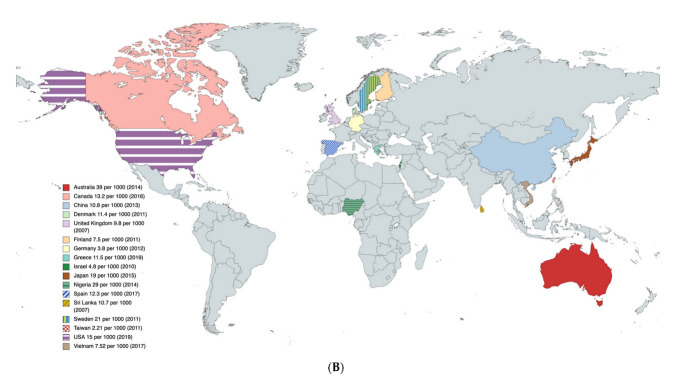
Prevalence (estimate per 1000) of autism spectrum disorders (ASD) in the world. (**A**) Prevalence trend for select countries; (**B**) Worldwide prevalence by year. Created with Mapchart.net.

The worldwide prevalence of ASD is estimated to be close to 1.5% [2,3,4,5]. ASD prevalence varies depending on the year and the country dataset. Figure 1 was designed using the results of existing ASD datasets (https://data.cdc.gov/Public-Health-Surveillance/autism-prevalence-studies/9mw4-6adp, accessed on 20 April 2022). Figure 1A shows that, in general, the highest prevalence was observed in Australia (39 per 1000), followed by Sweden (21 per 1000) and Japan (19 per 1000), and a lower prevalence was observed in Taiwan (2.21 per 1000). In Figure 1B, it can be seen that prevalence has been increasing recently in some countries, such as Australia (from 3.92 per 1000 in 2002 to 39 per 1000 in 2014), Japan (from 1.55 per 1000 in 1983 to 19 per 1000 in 2015), the USA (from 0.33 per 1000 in 1985 to 15 per 1000 in 2019), and Denmark (from 0.43 per 1000 in 1962 to 11.4 per 1000 in 2011). These data suggest that external factors such as mode of life and industrialization may have affected individual behaviours and could play a role in ASD occurrence. However, prevalence rates may vary because of differences in diagnostic procedures and reliability across countries, regions, and time frames.

#### 1.1.3. Signs and Symptoms of ASD

Individuals with ASD often display difficulty with social life, restricted interests, and repetitive behaviours [6]. Nevertheless, they may also have strengths that are superior to a neurotypical person. Table 1 shows common behaviours reported in ASD patients.

#### 1.1.4. Causes and Risk Factors

Genetics are primarily involved in the vast majority of ASD cases [7]. However, nongenetic or “environmental” factors can increase the incidence of ASD in genetically predisposed individuals [8]. Understanding ASD risk factors can contribute to a better understanding of the biological basis of the disorder. Research has shown that an older parental age also contributes to a higher incidence of their child having ASD [9]. Similarly, having a sibling with ASD increases the incidence of developing ASD by 2–18% [10,11,12,13]. Studies have shown that among identical twins if one child has been diagnosed with ASD, there is more than a 36–95% chance that the second child will also develop ASD. In the case of nonidentical twins, the incidence is evaluated to be approximately 31% [10,11,14,15]. In addition, individuals with certain genetic conditions, such as Down syndrome or Fragile X syndrome, have a higher incidence of developing ASD [16,17,18]. These data suggest that ASD could be the result of disruptions in genetic factors during prenatal development. Furthermore, with the recent events of COVID-19, concerns exist about a possible connection between childhood vaccinations and ASD development. Nevertheless, studies seem to refute any link between vaccination and autism [19,20,21,22].

#### 1.1.5. ASD Diagnosis

**Figure 2:**
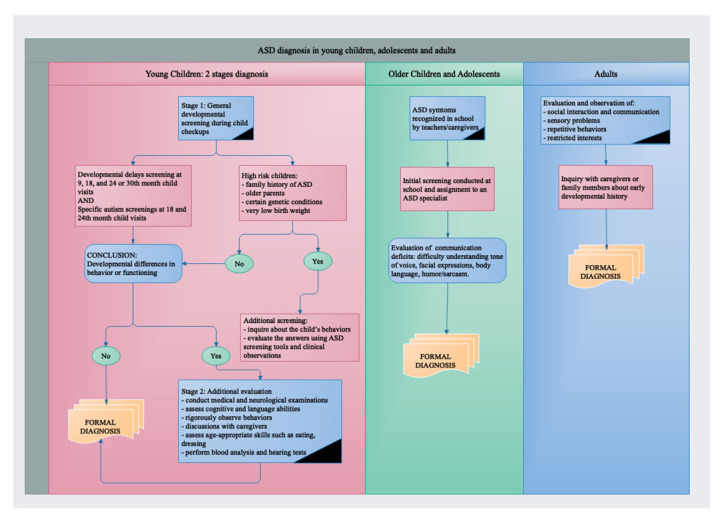
ASD diagnosis. In young children, diagnosis is conducted at 2 different stages (general developmental screening and additional evaluation). However, in adolescents and adults, diagnosis is more difficult, and symptoms are identified following performance in school or social behaviours.

ASD is diagnosed by evaluating behaviour and development. Although diagnosis can be performed at any age, it is better to diagnose ASD by the age of two, and treatments can be started earlier for better efficacy [6,23,24]. Figure 2 presents a summary of ASD diagnosis, depending on the developmental stage.

#### 1.1.6. Treatments of ASD

Once the diagnosis is confirmed, ASD treatment should begin immediately because treatment timing could impact the outcome [6]. The difficulty with ASD is that there is no proper best treatment, as individuals face different kinds of diverse and complex symptoms. Other reports have described the principal medications adapted for ASD. Principally, medications can help with the symptoms of irritability, aggression, hyperactivity, attention, and repetitive behaviour. In addition, behavioural, psychological, and educational programs involving specialists, caregivers and trusted family members are reported to help improve social, communication, and language skills [25,26]. These therapies also have the advantage of reducing abnormal behaviours and increasing the life skills necessary to overcome the disorder. Of particular interest, depending on the age and the individual, cognitive behavioural therapy (CBT) modifications can help. CBT approaches are performed to teach individuals how to monitor their feelings and perceptions. CBT targets both cognition (thinking) and behaviour (action) and is used as a therapeutic intervention for individuals with anxiety and depression [27,28].

### 1.2. Overview and Epidemiology of Attention-Deficit/Hyperactivity Disorder (ADHD)

#### 1.2.1. Overview

Attention-deficit/hyperactivity disorder (ADHD) is a particular disorder marked by an alternation of inattention and/or hyperactivity-impulsivity that interferes with functioning or development. People with ADHD face an ongoing pattern of:

- Inattention: difficulty doing a task and staying focused and organized.
- Hyperactivity: moving constantly, including in inappropriate situations, or demonstrating excessive fidgets, taps, or talks. In adults, hyperactivity is manifested by extreme restlessness or talking too much.
- Impulsivity: acting without thinking or difficulty with self-control. Importantly, it can be manifested by a desire for immediate rewards or the incapacity to wait for gratification.

#### 1.2.2. Prevalence

**Figure 3:**
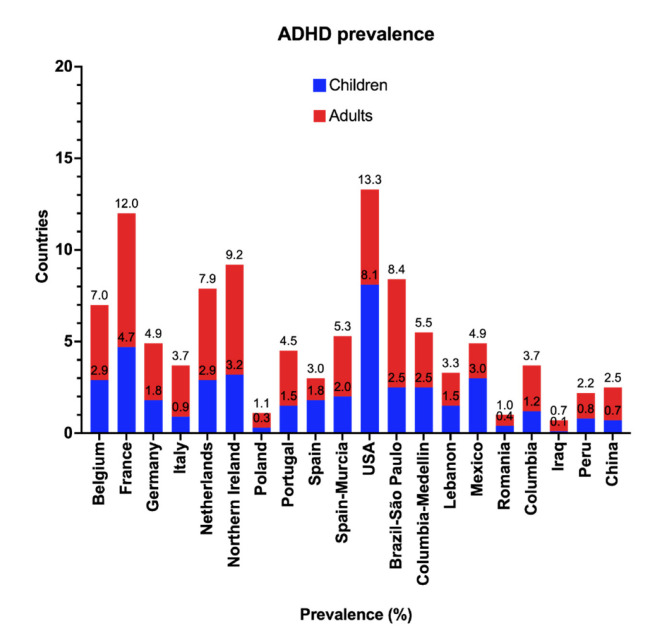
Overall prevalence of ADHD in children vs. adults in select countries in 2017. Prevalence varies depending on the country [29,30].

ADHD prevalence can be classified into two types: among children and adolescents and among adults. The average worldwide prevalence of ADHD is ~2.2% overall (range, 0.1–8.1%) in children and adolescents (aged < 18 years). From a range of countries in Asia, Europe, the Americas and the Middle East, the prevalence is ~2.8% overall (range, 0.6–7.3%). Worldwide prevalence data on ADHD in children are scarce; however, country-specific prevalence studies have been conducted all over the world. As demonstrated in Figure 3, the highest prevalence in 2017 was in the USA (8.1%), whereas the lowest was reported in Iraq (0.1%), Poland (0.3%) and Romania (0.4%). Similarly, in adults, the lowest prevalence was in Iraq and Romania (0.6% each), and the highest prevalence was reported in France (7.3%) [29,30]. As was observed for ASD prevalence, the variability in ADHD prevalence data may be due to diagnostic procedures and genetic and environmental factors.

#### 1.2.3. Signs and Symptoms of ADHD

ADHD symptoms are expressed either by inattention or by hyperactivity-impulsivity. However, both types of symptoms can be observed in the same individual. These symptoms can be severe and impede social activity at school, work or in general life. Table 2 describes the symptoms of individuals with ADHD and their different components.

#### 1.2.4. Diagnosis of ADHD

In general, central nervous system (CNS)-related disorders such as stress, sleep disorders, anxiety and depression can cause symptoms like those of ADHD. Therefore, the diagnosis must be pronounced by having chronic or long-lasting symptoms of inattention and/or hyperactivity-impulsivity. In addition, these symptoms must be troublesome to the person’s activities and lead them to fall behind typical development for their age. ADHD symptoms are first observed at the ages of 3–6 years old and can persist through adolescence and adulthood or change as the individual grows up. Hyperactivity-impulsivity is the most predominant symptom in childhood, and with age, the symptom of inattention may become more prominent. In contrast, during adolescence, hyperactivity starts to lessen, but inattention and impulsivity may remain. Inattention, restlessness, and impulsivity are reported to persist into adulthood [31].

#### 1.2.5. Risk Factors

Like ASD, the causes of ADHD are not well understood. However, the genetic component seems to play a major role. In addition, environmental factors such as strong stimuli, brain injuries, nutrition, and social factors might play a role in ADHD. It is important to note that ADHD is more frequent in boys than in girls, and girls are more likely to demonstrate inattention symptoms. Learning disabilities, anxiety disorder, conduct disorder, depression, substance abuse, and early life exposure to chemicals such as lead or nicotine from tobacco are often associated with ADHD [32,33].

#### 1.2.6. Treatment of ADHD

To date, there is no cure for ADHD, but the symptoms can be managed with adequate treatment. Current therapies include medication, psychotherapy, education, or a combination of treatments. Medication includes the use of stimulants that increase dopamine and norepinephrine levels in the brain or non-stimulants that can also improve focus, attention, and impulsivity. Antidepressants are sometimes used alone or in combination with a stimulant to treat ADHD.

Psychotherapy including only individual treatment sessions with the child (without parent involvement) is reported to not be effective for managing ADHD symptoms and behaviour. This demonstrates that in addition to psychotherapy, parents and family participation are important. Parents must reward positive behaviours, encourage behaviour changes, and improve interactions with the person with ADHD. Similarly, ignoring or redirecting the child’s inadequate behaviours can help improve symptoms.

### 1.3. Key Differences, Similarities and Conditions That Can Be Mistaken for ASD or ADHD

ASD and ADHD share several similarities and differences, as presented in Table 3. In the same way, several conditions exist that may be confused with or appear along with ASD or ADHD. It is important to discriminate against these conditions for appropriate treatment. More, as described further in Section 3, researchers should be careful when using animal models to investigate ASD or ADHD, as these models must specifically demonstrate face validity, construct validity and predictive validity, to be accepted.

### 1.4. Molecular Biology and Mechanisms Underlaying ASD and ADHD

#### 1.4.1. ASD

The dopaminergic system is mainly implicated in the neuropathology of ASD. In the limbic and cortical brain which controls locomotion, emotion, cognitive and endocrine functions, the rs6280 (C>T) SNP of the first dopamine receptor D3 exon results in a serine to glycine substitution (ser9Gly), leading to mental disorder [34]. More, the glutamate metabotropic receptor 7 (*GRM7*) has spatiotemporal expression in the cerebral cortex, cerebellum, and hippocampus [35], and an rs779867 (T>G) is an intronic polymorphism of *GRM7* strongly associated with ASD in children [36]. Similarly, recent evidence suggested that the rs849563 (T>G) polymorphism located at exon 10 of neuropilin-2, the non-tyrosine kinase cell surface glycoproteins, could contribute to ASD [37].

In addition, nicotinamide adenine dinucleotide (NADH) oxidase activity is suggested to be implicated in ASD pathology. Indeed, reduced levels of NADH were reported in lymphocytic mitochondria, whereas plasma pyruvate levels were elevated in ASD children [38]. 

Tumour necrosis factor (TNF-*α*), an inflammatory cytokine produced by macrophages and monocytes, is solicited during acute inflammation to mediate cell proliferation, differentiation, and apoptosis [39]. Higher levels of TNF-*α*, IL-6, and IL-17 and lower levels of IL-2 were observed in ASD patients [40]. 

In addition, vesicular monoamine transporter 1 (*VMAT1*) has a role in the accumulation of cytosolic monoamines into synaptic vesicles and is implicated in the mechanistic of anxiety, affective, and alcohol addiction disorders. It has been demonstrated that rs1390938 G/A genotype polymorphism of *VMAT1* is significantly correlated with ASD [41]. 

Furthermore, vitamin D3 deficiency during pregnancy and early childhood was suggested to be implicated in the progression of ASD [42]. Furthermore, the rs16976358 T>C polymorphism of GTP-binding protein RIT2 is reported to play a role in ASD, Alzheimer’s disease, schizophrenia, and bipolar disorder [43].

#### 1.4.2. ADHD

Two major pathways have been proposed to address the molecular biology of ADHD: dysfunctions in the dopaminergic-fronto-striatal pathway and alterations of the circadian rhythm. There is ample evidence of the implication of the dopaminergic system in the physiopathology of ADHD [44]. Although neurotransmitters such as norepinephrine are also implicated, only the dopaminergic system has been extensively explored [45]. As a matter of fact, methylphenidate, a potent selective dopamine reuptake inhibitor, is a common medication used in ADHD treatment. Neurobiology and imaging systems have tried to delineate ADHD mechanisms; however, ADHD behaviours are also manifested in several disorders, making it difficult to clearly explain it. Recently, molecular imaging has been suggested to be useful in understanding the molecular pathophysiology of ADHD [46]. The dopamine reuptake transporter (DAT) located in the striata is the site of action of methylphenidate and allows the reduction of dopamine synaptic concentration [47]. By using molecular imaging, scientists have suggested that an increase in DAT protein expression in the striata due to genetic or environmental factors, could principally lead to ADHD [48]. However, contradictory results exist to refute that hypothesis, confirming again the variability and inconsistency of results to explain ADHD molecular physiopathology [49]. Further, it has been reported a high level of D2 and D3 receptors in patients with a history of perinatal cerebral ischemia and ADHD symptoms. A mechanism of upregulation in postsynaptic D2/D3 receptors was explained by loss in dopamine neurons due to the ischemia or an increase in presynaptic dopamine reuptake [50]. Although dysfunction of the infraorbital prefrontal cortex is implicated, the molecular biology of ADHD remains unclear.

A disturbed circadian rhythm and altered sleep are also key features of ADHD [51]. Circadian rhythm is driven by circadian locomotor output cycles kaput (*CLOCK*) genes, which regulate several factors such as weight and mood, as demonstrated by obesity or mood and CNS-related disorders occurring when these genes are altered. Similarly, ADHD medications are known to improve sleep efficacy and alter *CLOCK* genes’ expression [52]. At a molecular level, circadian rhythm is assured by several transcription-translation feedback loops controlling the expression of *CLOCK* genes [53]. There is a recent accumulation of evidence of circadian rhythm implication in ADHD molecular mechanisms. For example, *CLOCK* genes aryl hydrocarbon receptor nuclear translocator-like (*BMAL1*) and period circadian regulator 2 (*PER2*) showed circadian rhythmicity in control patients whereas ADHD patients showed inconsistency in addition to significant phase delayed cortisol rhythms [51]. Although there is evidence that the dopamine system and circadian rhythm are fully implicated in ADHD, additional resources are needed to fully understand this disorder.

## 2. Current Behavioural Tests of ASD and ADHD in Research

For a long period of time, mice were the most used animal models in behavioural neuroscience research. Indeed, mice are mammalian species and can display a variety of behaviours that resemble human diseases. In addition, the emergence of tools for direct genome manipulation has allowed scientists to easily investigate the impact of genes on development and behaviour [54,55]. However, recently, zebrafish, or *Danio rerio*, a freshwater fish that inhabits rivers in several places in Asia, has gradually attracted the interest of scientists. Indeed, zebrafish have become one of the preferred in vivo model organisms for studying diverse human diseases regarding developmental conditions [56,57], embryogenesis, regeneration, and behaviour [58,59,60]. In particular, zebrafish offers many advantages in the field of neuroscience, such as good tractability, ease of genetic manipulation, and amenability to high-throughput screens. Furthermore, zebrafish embryos and larvae are transparent, making them an excellent system for analysing developmental processes and neural signalling in vivo.

Both mice and zebrafish present some advantages and limitations, and it is important to take them into consideration in the field of neuroscience. In Table 4, we compare these two species closely related to humans.

A valid model for any human disorder, including ASD or ADHD, should demonstrate the following capabilities [61]:

- Strong similarity to human phenotype.
- Same biological phenomena that are responsible for the disease in humans.
- Similar response to potential treatments used in humans.

Mice and zebrafish have demonstrated their importance as animal models in neuroscience. They validate the requirements for a valid model in evaluating ASD or ADHD disorders, as demonstrated by several studies of their use as animal models in the core areas affected in ASD and ADHD patients. Table 5 presents a summary of important behavioural tasks in mice and zebrafish to investigate ASD and ADHD.

The three core areas affected in individuals with ASD are socialization, nonsocial patterns of behaviour (repetitive behaviour, motor abnormalities and restricted activities) and communication [62,63], whereas the affected core areas in individuals with ADHD are hyperactivity or impulsive behaviours, deficits in attention and memory problems, and aggressiveness [64,65]. Overlapping symptoms for both ASD and ADHD mainly include social problems, attention difficulty and speech/language delays.

**Table 5:** Behavioural tasks to investigate ASD or ADHD-like alterations in mice and zebrafish. Summary of some behavioural tests in mice and zebrafish relevant to ASD and ADHD.

Disorders	Core Areas Affected	Behavioural Tests	
		Mouse	Zebrafish
ASD	Socialization	Novel partner preference test/Social approach test [65,66,67,68] Reciprocal social interaction test [3] Juvenile play test [68]	Social preference test [69] Shoaling test [69] Social interaction test [70]
	Nonsocial behaviours (repetitive behaviour, motor alterations and limited range of activities)	Self-grooming test [66,67,68] Repetitive novel object test [71,72] Open-field test [65,68] Social transmission of food preference [68] Predator avoidance test	Open field test [73,74,75,76,77] T-maze test [77] Predator avoidance test [78,79]
	Communication	Social transmission of food preference test [68] Impaired vocalization test [80,81]	Not available to date
ADHD	Attention and learning deficits	Y-maze spontaneous alternation test [66] Barnes maze test [65,66]	Five-choice serial reaction time task (5-CSRTT) [82,83] T-maze test [77] Inhibition avoidance task [84]
	Hyperactivity-Impulsivity	Open field test [66]	Open field test [74,75,76] T-maze test [77] Five-choice serial reaction time task (5-CSRTT) [82,83] Novel tank test [85,86,87]
	Aggressiveness	Resident–Intruder Paradigm [65,88,89]	Mirror test [69,70,90]

### 2.1. Behavioural Tests in Mice

#### 2.1.1. The Social Approach Test

**Figure 4:**
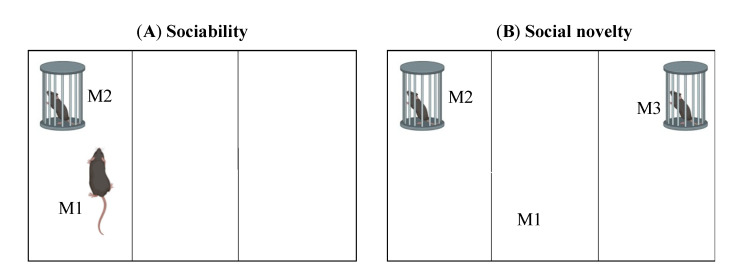
The social approach test. In the test for sociability (**A**), the test mouse, M1, chooses between spending time in the side with an unfamiliar mouse, M2, or in an empty side. In the test of preference for social novelty (**B**), the test mouse, M1, is now given a preference choice between the first mouse, M2, and a newly introduced unfamiliar mouse, M3. Created with Biorender.com.

The social approach test evaluates sociability and preference for novel society and is adapted to detect behavioural characteristics of ASD in mouse models [65,66,67,68]. As shown in Figure 4, the apparatus is usually made of Plexiglass and consists of three chambers (~20 × 40 cm per chamber). The test is performed in three phases. In phase 1, the three chambers are separated, and a mouse, M1, is placed in the middle of the chamber and allowed to habituate to the apparatus. After 5 min, a new mouse, M2, unfamiliar to mouse M1, is placed in a small metal cage and introduced into one of the two side chambers. An identical empty small metal cage is placed next to the adjacent side chamber, and the chamber separators are removed. In phase 2, mouse M1 is allowed to move freely for 10 min. The duration of time that it spends in the chamber containing mouse M2 and the duration of time it spends oriented towards the cage with its nose pointing less than 2 cm from it is recorded. In phase 3, a new mouse, M3, is placed into the previously empty cage. Mouse M1 is allowed to freely explore the apparatus for 10 min. The duration of time spent in contact or oriented towards the new cage containing mouse M3 compared to the cage with mouse M2 is also recorded [65,66,67,68]. A reduction in sociability time is associated with an ASD phenotype.

#### 2.1.2. The Reciprocal Social Interaction Test

After 30 min of acclimation to the experimental room environment, a mouse is placed in a cage containing fresh bedding. After 15 min of acclimation, a new mouse is added to the cage, and the two animals are allowed to freely interact for a period of 20 min. Behavioural events such as sniffing, following, grooming, mounting, huddling, and wrestling are recorded [68,69]. For the ASD phenotype, a reduction in social interaction is observed.

#### 2.1.3. Juvenile Play

Mice are brought to the testing environment a day before actual testing for habituation. Each test subject is then moved to individual cages with no access to food or water for 1 h. Each mouse is then deposited in the play testing arena for a 10-min habituation period. After all mice are habituated, each is replaced in its home cage with all cage mates. In the actual test, individual bouts and durations of social interaction parameters, including following, pushing past, crawling, nose-to-nose sniffing, anogenital sniffing and social grooming, are recorded [68]. Social interaction parameters are reduced in the ASD phenotype.

#### 2.1.4. Repetitive Grooming Test 

This is a simple test in which mice are first individually acclimated in a video-equipped cage for 10 min. Following the habituation period, the number of grooming sessions and total time spent grooming are determined by video surveillance at 10, 15, and 20 min [66,67,68]. An increase in grooming sessions is associated with ASD behaviours.

#### 2.1.5. Repetitive Novel Object Test 

**Figure 5:**
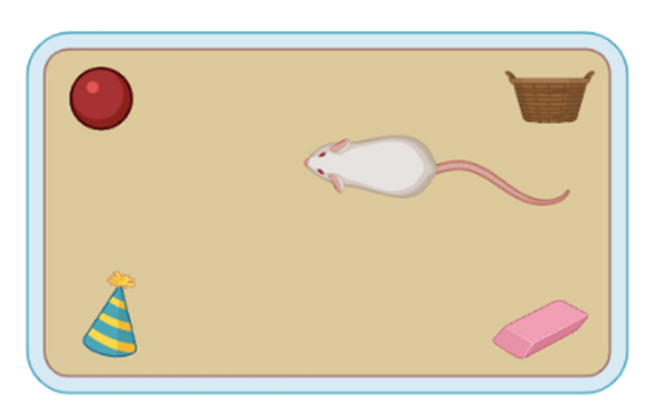
A mouse with four novel objects for the repetitive novel object contact task. Small toys/objects are used to evaluate mouse repetitive behaviour. Created with Biorender.com.

In the repetitive novel object test (Figure 5), mice are evaluated for the frequency of repetitive contact with novel objects. On Day 1 of the test, mice are introduced to the experimental room and left undisturbed for a 30 min habituation period. The next day, the animals are individually placed in an identical clean cage containing fresh sawdust bedding as well as four novel objects (small children’s toys) located approximately 4 cm from each of the four corners. Close contact with or burying of the novel objects is recorded during a 10-min session test. The occurrence of repetitive contact with three and four toys, the frequency of times that the mice buried each object, the total frequency of contact with each of the toys, and the total number of burying episodes are calculated [71,72]. Restricted and increased repetitive behaviours are a core feature of ASD.

#### 2.1.6. The Social Transmission of Food Preference 

**Figure 6:**
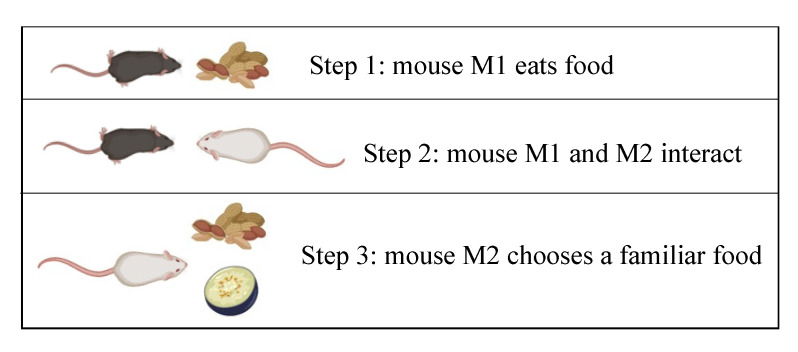
The social transmission of food preference. A classical test to evaluate social transmission of food preference between two mice; mouse M1 encounters the food and transmits information to mouse M2. Created with Biorender.com.

The social transmission of food preference test is used to investigate the communication of information obtained through social interactions. As demonstrated in Figure 6, mice can communicate and overcome their avoidance of a novel unfamiliar food by sniffing the mouth, face and whiskers of another mouse. In step 1, a mouse M1 is given the new food. In step 2, by socially interacting with that mouse, a different mouse M2 obtains information regarding the food, and in step 3, the first mouse is fasted overnight and given access to a new food with different flavours. The two mice are then left together for a period of 10 min. Afterwards, the second mouse is fasted overnight and is given a choice between the two flavoured foods for the preference choice task [68].

#### 2.1.7. The Resident–Intruder Paradigm 

The test is performed to assess territorial aggression on five different days. On each testing day, an unfamiliar intruder mouse is randomly assigned to a mouse (resident) for interaction. The housing cage of the resident mouse is used as the interaction area. A transparent Plexiglas separator that can enable visual, auditory, and olfactory perception is placed in the middle of the cage to prevent direct interaction between animals. The intruder mouse is placed on the other side of the plastic screen for a period of 5 min. The separator is then removed, and the interaction is recorded for 5 min. The frequency of attacks and bites and the latency to the first attack, the number of lateral threats, and tail rattles are analysed for all interaction days [65,88,89].

#### 2.1.8. The Y-Maze Spontaneous Alternation Test 

**Figure 7:**
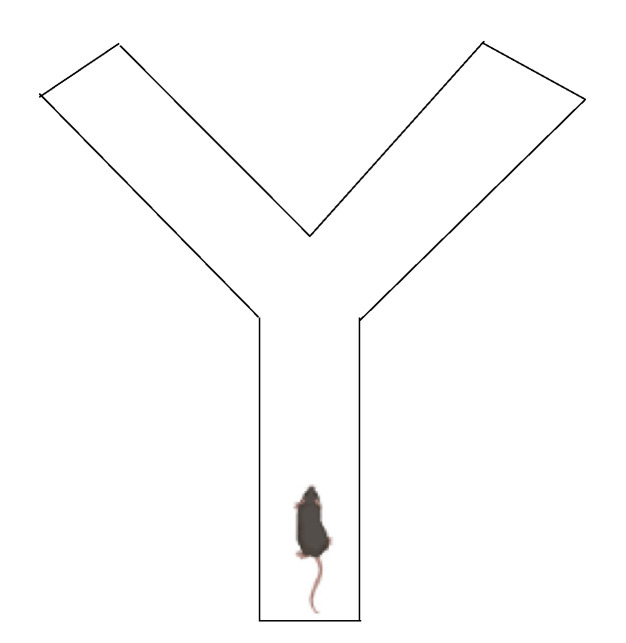
The Y-maze spontaneous test in mice. Working memory is evaluated as in the Barnes maze test.

This behavioural test is used to assess working memory (Figure 7). Spontaneous alternation can be evaluated by individually placing animals in one arm of a symmetric Y-maze made of opaque black acrylic walls and recording the sequence of arm entries and the total number of entries over an 8-min period session. This test evaluates the tendency a mouse has towards choosing a distinct path from the one it previously chose (deemed spontaneous alternation) and hence requires memory of its previous choice [66]. Deficits in spontaneous alternations are characteristic of the ADHD phenotype. However, this test is not as definitive as the Barnes maze and other more specialized learning/memory tests.

#### 2.1.9. The Barnes Maze Test 

**Figure 8:**
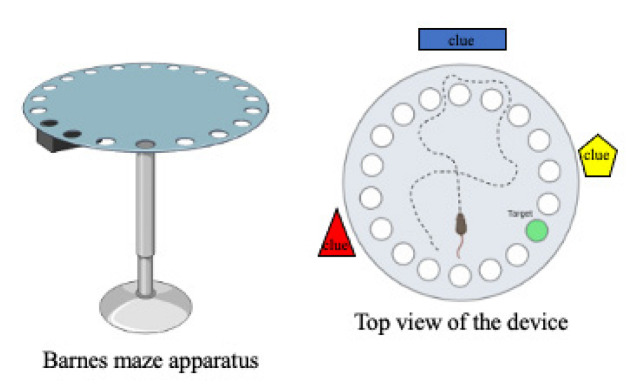
The Barnes maze test in mice. Different cues are given to the mouse to find an escape in the Barnes maze apparatus. Created with Biorender.com.

The Barnes maze test is a spatial-learning task that allows mice to use spatial hints to locate a way of escaping from a mildly aversive environment. In this test (Figure 8), mice can be assessed for their ability to learn the location of an escape box over the course of 9 days in the Barnes maze apparatus. The escape hole is constant for each mouse over 5 training days, and each mouse is then tested three times per day for 4 days, followed by no testing for 2 days and retesting on Day 7 [65,66]. Inattention and memory impairment are typically observed in ADHD phenotypes.

#### 2.1.10. The Impaired Vocalization Test 

Ultrasonic vocalizations are recorded in mice after an experiment using a male subject and female urine exposure [81,82]. Briefly, vocal emissions and acoustic data during the 5 min female urine exposure are recorded, and an observer counts the number of ultrasonic vocalizations emitted during the 5 min female urine exposure. In addition, the number of ultrasonic vocalizations emitted during the first 3 min of female urine exposure as well as their numbers in 10 s time bins is determined, to evaluate the time course of the ultrasonic vocalization response [80,81]. Reduced ultrasonic vocalizations are observed in mice with ASD phenotypes.

### 2.2. Behavioural Tests in Zebrafish

#### 2.2.1. The Social Preference Test 

**Figure 9:**
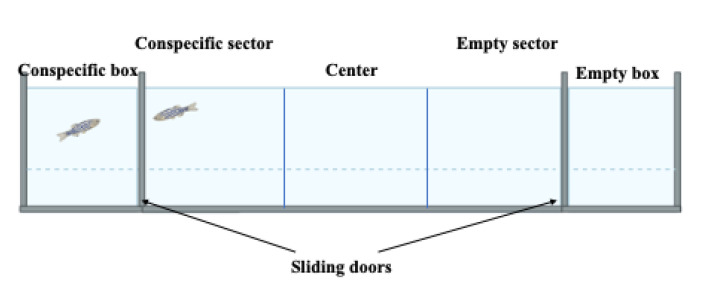
Social preference test apparatus in zebrafish. This test is ideal for investigating social behaviour in zebrafish. Created with Biorender.com.

Like the mouse social preference test, this test evaluates zebrafish social behaviour and locomotor activity (Figure 9). Briefly, a target conspecific fish is introduced to a conspecific compartment, separated by transparent sliding doors from the rest of the apparatus. Zebrafish are individually introduced to the central arena, which is separated by sliding doors from the two arms of the corridor. After a short period of time, the sliding doors are removed, and zebrafish can freely explore the apparatus for 6 min. Their behaviour is recorded, specifically the number of centre entries, time spent in the centre, the number of “conspecific” arm entries, the number of “nonconspecific” (empty) arm entries, total arm entries, and time spent in the respective zones of the apparatus [77]. Like the test in mice, reduced social preference is indicative of an ASD phenotype.

#### 2.2.2. Shoaling Test 

**Figure 10:**
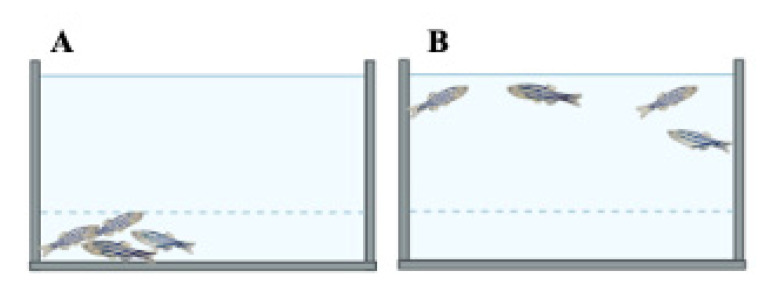
Shoaling test in zebrafish. (**A**) Normal shoaling behaviour; (**B**) disrupted shoaling behaviour. Created with Biorender.com.

Zebrafish are shoaling animals and are observed in groups for their shoaling behaviour. In this test, the distances (cm) between each fish in the group and the average interfish distance are recorded after an observation period of 20 min (Figure 10) [77,91]. Reduction in shoaling behaviour is usually likened to the altered social interaction in ASD phenotypes.

#### 2.2.3. Five-Choice Serial Reaction Time Task (5-CSRTT)

**Figure 11:**
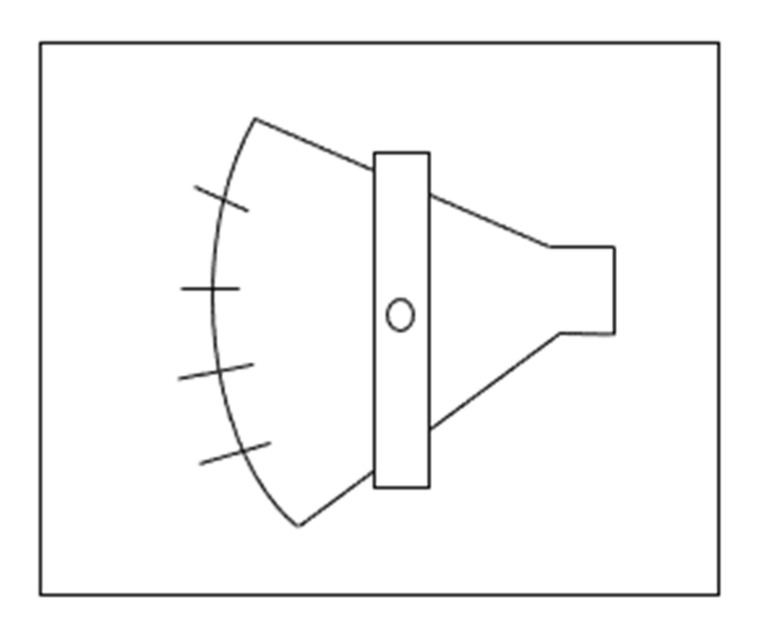
Schematic representation of the 5-CSRTT apparatus. The test is adapted from the rodent version.

This test investigates impulsiveness and attention by measuring the ability of zebrafish to respond to one of five perceptually identical stimuli that are applied randomly after a variable intertrial interval. It was adapted from the 5-CSRTT in rodents (typically the rat or mouse) and requires the animal to correctly identify which of the five apertures has been briefly illuminated to receive a reward. The results of this test in zebrafish are demonstrated to parallel those in mammals (Figure 11) [82,83].

#### 2.2.4. The Novel Tank Test 

**Figure 12:**
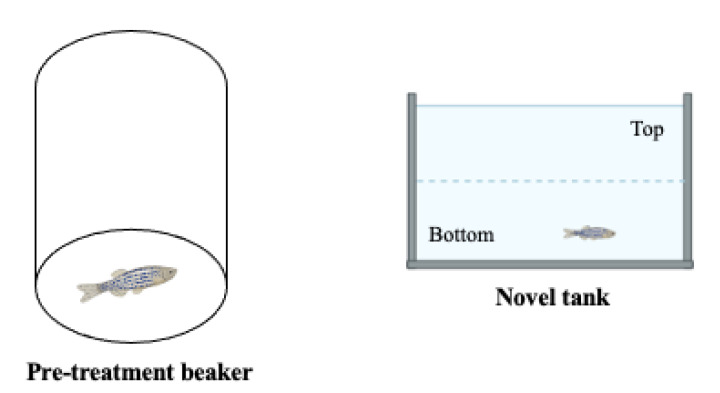
The novel tank diving test. Zebrafish are first exposed to a pretreatment beaker before being moved into the novel tank for behavioural observation and phenotyping. Created with Biorender.com.

This is the most commonly used test to assess locomotion and anxiety-like phenotypes, as the recorded parameters can be used to assess hyperactivity [86,87,88]. After a pretreatment period in a beaker, zebrafish are introduced into a novel environment, where they usually swim in the bottom section and gradually increase their swimming activity in the upper sections of a tank. Total distance travelled, average speed, absolute turn angle and immobility time are important parameters recorded during the 6-min test session (Figure 12) [85,86,87]. In ADHD phenotypes, an increase in distance travelled and a decrease in immobility time are generally observed.

#### 2.2.5. The Mirror-Attack Test 

**Figure 13:**
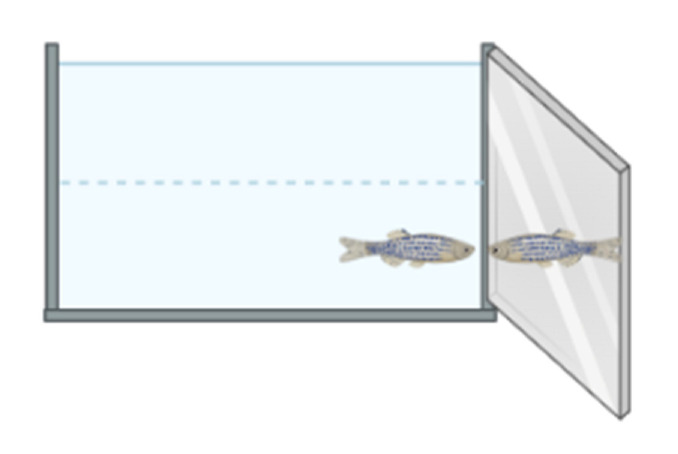
Mirror attack test in zebrafish. Zebrafish attack their own image, and aggressive behaviour is observed in ADHD individuals. Created with Biorender.com.

Zebrafish are placed in an experimental tank (L30 cm × H15 cm × W10 cm), and a mirror is placed at the side of the tank (Figure 13). Before the test, zebrafish are added to the tank and allowed to habituate for 60 s. The aggressive behaviours toward the zebrafish mirror image are then recorded over a period of 5 min. The tank is divided into four equal sections, and the number of entries and time spent in each section are recorded [69,70,90]. More aggressive periods, such as attacking one’s image in the mirror, are noted in zebrafish with the ADHD phenotype.

### 2.3. Behavioural Tests Common to Mice and Zebrafish

#### 2.3.1. The Open Field Test 

**Figure 14:**
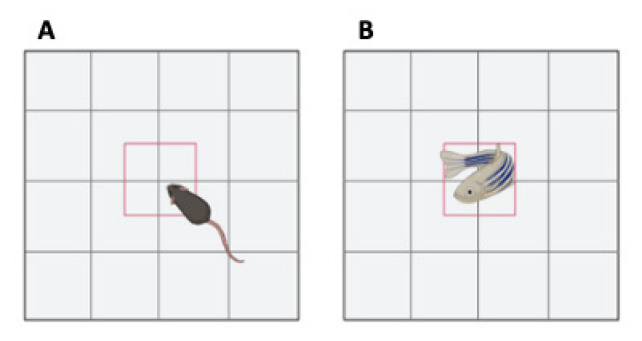
The open field test. (**A**) Test in mice; (**B**) test in zebrafish. An increase in locomotor/exploratory activity is characteristic of ADHD phenotypes. Created with Biorender.com.

The open field test is a well-recognized test to approach locomotor activity [73,74,75,76,77,92,93,94,95]. Multiple variants of the test exist for mice and depend on the parameters that are targeted by the operator. It consists of introducing the mice in an open field apparatus made in a Plexiglas box and measuring mouse movements during a fixed period (5–60 min) [93,95,96] (Figure 14A).

In zebrafish, locomotor activity can be assessed in larval zebrafish by placing animals in well plates and recording for 5–10 min [74,75,76,77]. Swimming episode frequency and duration, swim speed, active swim time and total distance swum are measured (Figure 14B).

#### 2.3.2. The Predator Avoidance Test 

**Figure 15:**
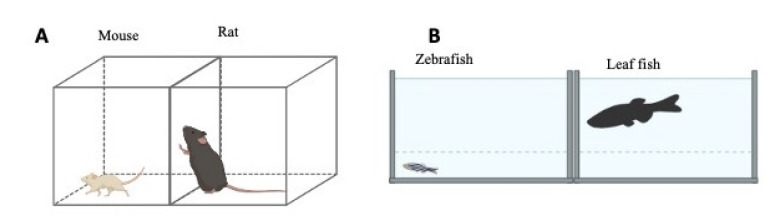
The predator avoidance test. (**A**) Test in mice; (**B**) test in zebrafish. Created with Biorender.com.

In this test, a natural predator of zebrafish or mice is introduced into a tank/cage well known for a given subject, and the following parameters related to avoidance and fear are recorded: distance between predator and test subject, predator approaching time, geotaxis, locomotor/swimming activity, turn angle, number of freezing episodes, and time spent frozen (Figure 15) [78,79]. In ASD, these parameters are modified to denote an increased avoidance of natural predators.

#### 2.3.3. The T-Maze Test 

**Figure 16:**
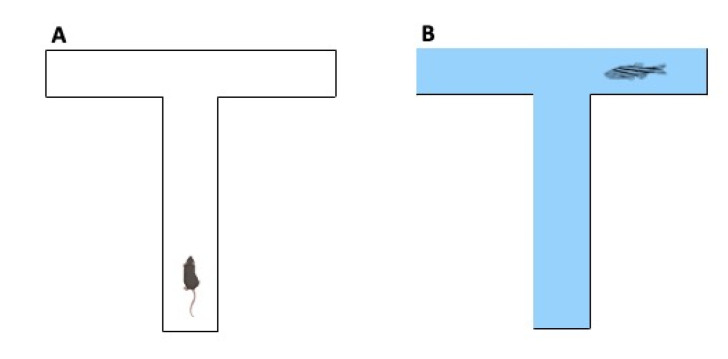
The T-maze test. (**A**) Test in mice; (**B**) test in zebrafish.

Spontaneous exploration of zebrafish or mice can also be assessed in the T-maze apparatus. In zebrafish, the device is a clear acrylic T-shaped box filled with water. Zebrafish or mice are introduced individually to the bottom arm of the T-maze (facing the wall) for a 6-min period. The number of centres and total arm entries and the number of freezing episodes and freezing duration are documented (Figure 16) [77]. Stereotyped behaviours and reduced exploratory activity are typical of ASD phenotypes.

## 3. Mouse Models of ASD and ADHD Research

The development of an animal model is a common approach to studying the mechanisms of a specific disease or disorder. Given the complexity of ASD and ADHD and their aetiology, genetic or pharmacological models are mainly used. Genetic models have recently been explored with the advent of new genetic tools, such as CRISPR/Cas9 gene-editing technologies. These tools can mimic either ASD or ADHD symptoms and reflect the neurobiology in animals. In general, animal models should mimic a clinical disorder as much as possible, with similar symptoms, treatment responses, and pathophysiology. More specifically, an adequate ASD or ADHD model should have three types of validity [64]:

- Face validity: mimic the fundamental behavioural deficits found in ASD or ADHD individuals;
- Construct validity: conform to the proposed pathophysiology or known therapeutics of ASD or ADHD;
- Predictive validity: predict unknown aspects of ASD or ADHD such as its genetics, neurobiology, or therapeutics.

### 3.1. Genetic Mouse Models of ASD

There are multiple genetic mouse models that can imitate ASD-like phenotypes in humans, and the majority of existing genetic models have been obtained by performing reverse genetics (alteration of the orthologous ASD-linked genes in mice) [61]. According to the SFARI GENE Database (https://gene.sfari.org/autdb/GS_Home.do, accessed on 10 May 2022), to date, more than 281 genes have been used to explore ASD phenotypes in mouse models [97]. We first discuss some of the most important or frequently reported models. A comprehensive list of mouse models of ASD candidate genes and the main phenotypes observed is presented in Table 6.

#### 3.1.1. Black and Tan Brachyury (BTBR) T+ tf/J Mice

This mouse strain has demonstrated social behaviour impairments such as reduced interaction, aversion for frontal interaction; communication impairments such as altered patterns; high levels of repetitive behaviours such as increases in self-grooming and persistent burying behaviours; and difficulties in learning-related tasks. BTBR T+ tf/J mice also show alterations in the development of the brain, and several ASD-linked genes have been identified to be disrupted in this strain [98,99].

#### 3.1.2. The Shank3 Knockout Mice

SH3 and multiple ankyrin repeat domains 3 (*Shank3*) is a postsynaptic density protein which plays a role in the structural and functional organization of the dendritic spine and synaptic junction [100]. *Shank3* includes an ankyrin repeat domain, a PDZ domain, and a Homer binding domain. Mutations can be inserted at each different domain, resulting in different impairments related to ASD phenotypes. For example, mice with a mutation in the ankyrin domain display impairments in excitatory neurotransmission and long-term potentiation; however, they show no trouble with sociability and only slight differences in ultrasonic vocalizations and repetitive behaviour [101,102]. Mice with a mutation in the PDZ domain display much more severe phenotypes, such as highly persistent self-grooming leading to skin lesions, impaired sociability, and reduced corticostriatal excitatory transmission [102]. When the mutation is at the Homer binding site, *Shank3* KO mice demonstrate more aggressiveness, reduced long-term potentiation, and enhanced long-term depression [103]. This strain is particularly interesting due to the diverse mutations at different sites resulting in different types of behaviours using the same gene.

#### 3.1.3. Fragile X Syndrome

Fragile X syndrome is one of the most frequent genetic causes of intellectual disabilities, and more than 30% of individuals with Fragile X syndrome meet the diagnostic criteria for autism [18]. Constriction at the end of the X chromosome is associated with a critical expansion of CGG triplet repeats, transcriptionally silencing the fragile X messenger ribonucleoprotein 1 (*Fmr1*) gene [104,105]. *Fmr1* is a multifunctional polyribosome-associated RNA-binding protein playing a central role in neuronal development and synaptic plasticity through the regulation of alternative mRNA splicing, mRNA stability, mRNA dendritic transport and postsynaptic local protein synthesis of a subset of mRNAs [100]. Mice with a mutation in *Fmr1* display impairments in long-term potentiation and abnormal social, cognitive, and anxiety-related behaviours [95,106,107,108]. The *Fmr1* mutation is also implicated in the upregulation of mGluRS receptors [109]. Interestingly, crossing mGluRS knockout mice with *Fmr*1 gene KO mice leads to rescue of the long-term depression and attenuation of seizures [110].

#### 3.1.4. The E3 Ubiquitin-Protein Ligase (*Ube3a*) Gene and 15q11-13 Duplication Maternal/Paternal

The 15q11-13 mutations are linked to duplication or gene deletion. For example, the loss of maternal genomic information at the 15q11.2-13 locus is responsible for Angelman syndrome, whereas paternal genetic material leads to Prader–Willi syndrome [111]. In individuals with ASD, maternal duplications and triplications of the 15q11-13 locus have been frequently observed. Interestingly, *Ube3a* is the sole gene expressed from the maternal allele in mature neurons, and its deletion or mutations are responsible for Angelman syndrome [112]. It serves as an E3 ubiquitin-protein ligase which accepts ubiquitin from an E2 ubiquitin-conjugating enzyme in the form of a thioester and transfers it to its substrates [100].

#### 3.1.5. The Contactin-Associated Protein-like 2 (*Cntnap2*) Gene

*Cntnap2* plays a role in the formation of functionally distinct domains critical for saltatory conduction of nerve impulses in myelinated nerve fibres [100] and its mutations are responsible for a syndromic form of ASD, cortical dysplasia, and focal epilepsy syndrome. The symptomatic form of the disorder consists of epileptic seizures, language regression, intellectual disability, and hyperactivity. KO mice for the mutation demonstrate autistic traits, a diminution of interneurons and abnormal neuronal network activity. However, abnormal behaviours are amended following risperidone administration [113]. It is important to mention that *Cntnap2* has also been found in a different mouse inbred strain located in divergent regions within the *C58*/*J* gene [114].

#### 3.1.6. Rett Syndrome

Methyl-CpG binding protein 2 (*Mecp2*) is a chromosomal protein that binds to methylated DNA [100]. Rett syndrome is caused by mutations in the *Mecp2* gene, and only affects girls, as it is linked to chromosome X [115]. The syndrome is characterized by intellectual disability, motor dysfunction, seizures, early death, and autism. Interestingly, KO of the *Mecp2* gene in males provokes a total loss of function, whereas, in females, there are evident ASD-linked behaviours [116]. In addition, loss of *Mecp2* from GABAergic neurons recapitulates different phenotypes such as repetitive behaviours, characteristic of Rett syndrome and ASD. More, *Mecp2* deficiency provokes a reduction in glutamic acid decarboxylase (Gad) 1 and 2 levels, suggesting that the implication of *Mecp2* in GABAergic neurons function is critical and that its mutation could alter GABA neurons and contribute to ASD [117]. It is noteworthy to mention that generation of a *Mecp2*-overexpressing mouse model or the use of an antisense oligonucleotide strategy successfully restored normal *Mecp2* levels and phenotype in *Mecp2* duplication adult mice [118].

**Table 6:** Mouse models of ASD and observed phenotypes. The main genes and corresponding phenotypes observed in ASD mouse models are presented.

Genes	Phenotypes	References
Actin like 6B (*Actl6b*)	Social and memory impairments, repetitive behaviours, hyperactivity	[119]
Activity dependent neuroprotector homeobox (*Adpn*)	Increased lethality, deficits in social memory, developmental alterations	[120,121,122]
Autophagy and beclin 1 regulator 1 (*Ambra1*)	Deficits in communication and social interactions, increased repetitive behaviours, reduced ultrasound communication in adults and pups, behaviour differences in male and female	[123]
Ankyrin repeat and sterile alpha motif domain containing 1B (*Anks1b*)	Social deficits, hyperactivity, and sensorimotor dysfunction	[124]
Rho GTPase activating protein 32 (*Arhgap32*)	Reduction in γ-aminobutyric acid type A receptor (GABAAR) levels and impaired GABAAR-mediated synaptic transmission	[125]
Rho guanine nucleotide exchange factor 10 (*Arhgef10*)	Impaired social interaction, hyperactivity, and decreased depression-like and anxiety-like behaviour	[126]
AT-rich interaction domain 1B (*Arid1b*)	Social behaviour impairment, altered vocalization, anxiety-like behaviour, neuroanatomical abnormalities	[127,128]
ASH1 like histone lysine methyltransferase (*Ash1l*)	Delayed eye development, increased lethality, infertility, dysfunction in immune response	[129,130]
ATPase phospholipid transporting 8A1 (*Atp8a1*)	Deficits in social behaviours	[131]
Ataxin1 (*Atxn1*)	Hyperactivity, impaired learning and memory, abnormal maturation and maintenance of upper-layer cortical neurons	[132]
Arginine vasopressin receptor 1B (*Avpr1b*)	Impaired social recognition, reduced pup ultrasonic vocalization	[133,134]
Cell cycle associated protein 1 (*Caprin1*)	Reduced sociality in a home cage and weak preference for social novelty	[135]
Coiled-coil and C2 domain containing 1A (*Cc2d1a*)	Reduced sociability, hyperactivity, anxiety, and excessive grooming	[135]
Chromodomain helicase DNA binding protein 2 (*Chd2*)	Developmental delay and increased mortality, decreased performance in object recognition test, reduced spatial working memory	[136,137]
Chromodomain helicase DNA binding protein 8 (*Chd8*)	Deficits in brain development, increased anxiety and repetitive behaviours, alteration in memory	[138,139,140,141,142]
Capicua transcriptional repressor (*Cic*)	Alteration in cortical and hippocampal morphology, reduced socialization	[143]
Contactin associated protein 2 (*Cntnap2*)	Delayed development, increased locomotor activity, impaired social interaction, and nest-building behaviours, increased epileptic behaviours	[144,145,146]
DEAD-box helicase 3 X-linked (*Ddx3x*)	Hyperactivity, anxiety-like behaviours, cognitive impairments in contextual fear memory but not novel object recognition memory, and motor deficits	[143]
Disco interacting protein 2 homolog A (*Dip2a*)	Excessive repetitive behaviours and defects in social novelty	[147]
DLG associated protein 1 (*Dlgap1*)	Post-synaptic density disruption and reduced sociability	[148]
Engrailed homeobox 2 (*En2*)	Reduced social interaction	[149,150]
Fibroblast growth factor 17 (*Fgf17*)	Reduced pup ultrasonic vocalization, lack of preference for social novelty, reduced reciprocal social interaction	[151]
Fragile X messenger ribonucleoprotein 1 (*Fmr1*)	Increased social approach, reduced repetitive behaviours, reduced anxiety, and normal locomotor activity	[108,152,153,154]
Forkhead box P2 (*Foxp2*)	Reduced pup ultrasonic vocalization, abnormality in Purkinje cells, severe motor impairments, premature death	[155,156,157]
Gamma-aminobutyric acid type A receptor subunit beta3 (*Gabrb3*)	Altered brain morphology, decreased sociability, reduced interneurons, increased seizures and anxiety, lack of preference for social novelty and impaired nest-building behaviour	[158,159,160,161]
Integrin subunit beta 3 (*Itgb3*)	Lack of preference for social novelty, and increased grooming behaviours	[162]
Lysine methyltransferase 5B (*Kmt5b*)	Deficits in neonatal reflexes and sociability, repetitive grooming, changes in thermal pain sensing, decreased depression and anxiety, increased fear, slower extinction learning, and lower body weight, length, and brain size	[163]
Methyl-CpG binding protein 2 (*Mecp2*)	Increased social avoidance, abnormal locomotor coordination, deficits in sociability and cognition	[116,164,165,166,167]
MET proto-oncogene, receptor tyrosine kinase (*Met*)	Deficits in cognitive function, hippocampal dysfunction	[168]
MicroRNA 137 (*Mir137*)	Dysregulated synaptic plasticity, repetitive behaviour, and impaired learning and social behaviour	[169]
Neuronal growth regulator 1 (*Negr1*)	Reversal learning deficits in the Morris water maze and increased susceptibility to pentylenetetrazol (PTZ)-induced seizures	[170]
Neuronal differentiation 2 (*Neurod2*)	Social interaction deficits, stereotypies, hyperactivity, occasionally spontaneous seizures	[171]
Neurite extension and migration factor (*Nexmif*)	Reduced sociability and communication, repetitive grooming behaviours, and deficits in learning and memory	[172]
Neuroligin 1 (*Nlgn1*)	Increased repetitive self-grooming, reduced pup ultrasonic vocalization, sociability, and reciprocal social interaction	[173,174,175,176]
Oxytocin receptor (*Oxtr*)	Impaired social behaviours, reduced pup ultrasonic vocalization	[177,178,179]
Protocadherin 19 (*Pcdh19*)	impaired behaviours including activity defects under stress conditions	[180]
Pogo transposable element derived with ZNF domain (*Pogz*)	Impaired social interaction	[181]
Phosphatase and tensin homolog (*Pten*)	High lethality, alteration in brain morphology, increased brain cells apoptosis, decreased Purkinje cells number, altered coordination and social memory and reduced sociability	[63,182,183,184,185]
RAB39B, member RAS oncogene family (*Rab39b*)	Cortical neurogenesis impairment and macrocephaly	[186]
Reelin (*Reln*)	Deficits in brain development, impaired coordination, and abnormal metabolism of neurotransmitters	[187,188]
Bifunctional polyamine/amino acid permease SAM3 (*Sam3*)	Impaired responses to social novelty, defects in social communication, and increased repetitive behaviour	[189]
Sodium voltage-gated channel alpha subunit 2 (*Scn2a*)	Increased cells apoptosis, seizures, hyperactivity, increased anxiety, and rearing	[190,191]
SUMO specific peptidase 1 (*Senp1*)	Social deficits and repetitive behaviours but normal learning and memory ability	[192]
SET domain containing 5 (*Setd5*)	Impairments in cognitive tasks, enhanced long-term potentiation, delayed ontogenetic profile of ultrasonic vocalization, behavioural inflexibility	[193]
SH3 and multiple ankyrin repeat domains 2 (*Shank2*)	Increased anxiety, hyperactivity, and repetitive behaviours, reduced social interaction and decreased social memory	[194,195,196]
SH3 and multiple ankyrin repeat domains 3 (*Shank3*)	Learning and sensory deficits, and impaired locomotor activity	[197]
TAO kinase 2 (*Taok2*)	Deficits in brain development, impaired memory, deficits in cortical layering, dendrite, and synapse formation, reduced excitatory neurotransmission and abnormalities in neural connectivity	[198]
T-box brain transcription factor 1 (*Tbr1*)	Increased anxiety and aggressiveness, reduced neural connections	[199,200]
Ubiquitin protein ligase E3A (*Ube3a*)	Low sociability, ultrasonic vocalization increased (pups) and decreased (adults) and impaired reversal learning	[201]
Urocortin 3 (*Ucn3*)	Abnormally low preference for novel conspecifics	[202]
UPF2 regulator of nonsense mediated mRNA decay (*Upf2*)	Impaired nonsense-mediated decay, memory deficits, abnormal long-term potentiation, increased social and communication deficits	[203]
UPF3B regulator of nonsense mediated mRNA decay (*Upf3b*)	Abnormal sleeping patterns, deficits in neural progenitors’ differentiation, impaired startle response	[204]

### 3.2. Pharmacological Mouse Models of ASD

Valproic acid (VPA) is an antiepileptic drug also used for the treatment of bipolar disorders, migraine, headaches, and neuropathic pain. VPA has teratogenic effects, including neural tube defects, cardiovascular anomalies, limb anomalies, and neurodevelopmental delay [205]. It is the major chemical used to induce ASD in mice, as demonstrated by behavioural abnormalities similar to those observed in autistic patients [115,206,207]. In addition, neuroanatomical and cellular changes similar to those in human ASD are observed in rodents exposed to VPA [208]. Other antipsychotic drugs have also been used, such as the glutamatergic antagonist phencyclidine (PCP; Table 7).

### 3.3. Genetic Mouse Models of ADHD

#### 3.3.1. The Dopamine Transporter Knockout Mouse (DAT-KO)

The dopamine transporter (DAT) is a presynaptic plasma protein found on dopaminergic nerve terminals that terminate dopamine signalling by rapidly sequestering dopamine released into the synaptic cleft [215,216]. The DAT-KO mouse demonstrates behavioural characteristics of ADHD, such as spontaneous hyperactivity and deficits in spatial memory [64,217,218]. The knockdown of DAT is responsible for a decrease in dopamine; nevertheless, dopamine metabolites, such as homovanillic acid (HVA), are increased, whereas the 3,4-dihydroxyphenylacetic acid (DOPAC) concentration does not vary [219]. In DAT-KO mice, a decrease in D1 and D2 receptors with approximately 50% decreases in both their mRNA and protein levels in basal ganglia have been previously reported [220,221]. DAT-KO mice also demonstrate important cognitive impairment in the eight-arm radial maze, a test of spatial learning [217,222]. Interestingly, stimulants such as amphetamine, methylphenidate, and cocaine can inhibit the hyperactivity observed in DAT-KO mice, but dopamine levels in the neostriatum were not increased when mice were administered these drugs [217,219,220,222]. Fenfluramine and quipazine (5-HT agonist) and fluoxetine (SRI antidepressant) also antagonize the hyperactivity observed in DAT-KO mice [222]. It is important to note that this mouse model also presents with abnormalities such as premature death or growth retardation, making it a less useful model. Indeed, no more than 68% of the homozygotes survived by week 10, and female mice show impaired maternal behaviour [220,223].

#### 3.3.2. Coloboma Mutant Mouse

The coloboma mutant (Cm) mouse was developed by application of neutron irradiation, and the heterozygote Cm^+/−^ mouse, which is the only viable strain, shows a variety of defects that resemble core features of ADHD. It shows, for example, delayed neurodevelopment and behavioural deficits such as hyperactivity and impulsivity [224,225,226,227]. Treatment with low doses of d-amphetamine (2–4 mg/kg) reduces hyperactivity, whereas methylphenidate (2–32 mg/kg) increases hyperactivity [225]. In addition, this mouse model has a mutation in the synaptosome-associated protein 25 (SNAP25) gene, and the authors suggest that the behavioural performance of coloboma mice could be related to SNAP25 dysfunction [225,228]. Further investigations are needed to better understand the mechanisms.

#### 3.3.3. Acallosal Mouse Strain I/LnJ

The I/LnJ mouse shows total callosal agenesis with complete penetrance, with behaviours such as learning deficits and hyperactivity, a reduced number of brief stops and a decrease in habituation in an open field [96,229]. Behavioural hyperactivity in this callosal agenesis model was demonstrated to be related to functional dominance of the right hemisphere because of the lack of callosal connections. However, neurotransmitter activity in this model remains unstudied.

#### 3.3.4. The Thyroid Hormone Receptor Beta 1 (*Thrb1*) Transgenic Mouse

This model of ADHD carries a mutant human *Thrb1* gene linked to human resistance to thyroid hormone (RTH) syndrome [230]. *Thrb1* is a nuclear hormone receptor having a high affinity for thyroid hormones and can act as a repressor or activator of transcription [100]. The *Thrb1* transgenic mouse shows hyperactivity; however, it does not demonstrate impulsivity or signs of inattention [231]. Nevertheless, a promoter for the *Thrb1* gene was used to demonstrate impulsivity, inattention, and hyperactivity in these mice [232]. The authors also suggested that an elevated dopamine turnover in these mice could be related to the catecholaminergic system [232]. It has been demonstrated that abnormal thyroid hormone levels can cause negative effects on brain development and cognition [233]. However, it is important to mention that the role of the thyroid system in ADHD remains unclear.

#### 3.3.5. *α*-Synuclein-Deficient Mice

The synucleins consist of a family of three proteins (*α*, *β*, *γ*), which are mainly present in presynaptic terminals [234,235,236]. The *α*-synuclein protein was demonstrated to be involved in the pathogenesis of Parkinson’s disease [237,238,239,240], suggesting that α-synuclein is important in the regulation of dopamine. Interestingly, mice lacking *α*- and *γ*-synuclein demonstrate signs of hyperactivity [241], which is associated with an increase in dopamine release.

### 3.4. Pharmacological and Environmental Mouse Models of ADHD

#### 3.4.1. Juvenile Mouse with a Neonatal 6-Hydroxydopamine-Induced Brain Lesion

A polymorphism of the D4 receptor has been linked to ADHD [242,243,244,245]. To investigate the role of the D4 receptor in ADHD, mice with neonatal 6-OHDA lesions can be useful because of the lack of D4 receptor activity. However, they do not show hyperactive behaviour compared to the wild type [246].

#### 3.4.2. Exposure to Chemicals

Exposure to environmental toxins such as lead and polychlorinated biphenyls (PCBs) or to pharmacological agents such as nicotine might lead to ADHD [32,247,248]. Mice exposed to lead from birth demonstrated important levels of spontaneous motor activity that were reduced by treatment with amphetamine and methylphenidate [249,250]. Analysis of forebrain tissue of mice following early life exposure to lead demonstrated an increased high-affinity transport of L-tyrosine and a decreased uptake of choline and dopamine. However, tissue levels of acetylcholine and dopamine were not increased [249,250]. Similarly, mice that were exposed to nicotine showed a full range of ADHD-associated behavioural phenotypes, including working memory deficits, attention deficits and impulsive-like behaviours. Nevertheless, the mechanisms and genes implicated have not been fully investigated [251,252,253].

#### 3.4.3. Maternally Stressed Mice

Adult offspring of mice treated with restraint stress during pregnancy have been shown to be hyperactive [254]. Furthermore, wheel-running activity is increased in these mice even after three days of habituation, and dopamine antagonists reduce this activity. Additionally, studies suggest a correlation between stress during pregnancy and ADHD [255,256]. However, the mechanisms remain unexplained.

## 4. Zebrafish Models of ASD and ADHD in Research

### 4.1. Genetic Zebrafish Models of ASD

One useful aspect of zebrafish is the ease of genetic manipulations on embryos and larvae. The most used zebrafish models consist of morpholino-based (MO) expression silencing. MOs are small modified oligonucleotides that can bind a selected target by complementarily knocking down gene function without altering its sequence [61,257]. Several examples of morpholino-based studies for ASD candidate genes exist and are presented in Table 8. However, MOs are effective temporarily, up to 4 days post fertilization (dpf), which does not allow the study of gene function beyond the early life of zebrafish [258]. Moreover, they can lead to off-target effects, resulting in nonspecific phenotypes for the gene of study [259,260]. Furthermore, targeted induced local lesions in the genome (TILLING) is a technique that has also been used. This technique is based on exposure to ethylnitrosourea (ENU), an alkylating mutagen that induces error-prone replication and leads to random point mutations in the genome. Sequencing is then performed to identify loss-of-function mutations. Several zebrafish ENU-KO models for ASD exist and are also presented in Table 8. Last, nuclease-based technologies, such as transcription activator-like effector nucleases (TALEN) and zinc-finger nucleases (ZFN), were recently established to improve the generation of novel zebrafish lines (Table 8) [261,262]. TALENs have a broader target potential but are restricted to simple mutations [263,264]. More recently, CRISPR/Cas9 technology has allowed the development of interesting zebrafish models (Table 8) [265,266,267].

The use of mutant zebrafish to study gene function is a challenging task because gene loss-of-function manipulations may leave significant amounts of mRNA produced by the targeted gene [294]. To facilitate the task for researchers, the Simons Foundation for Autism Research Initiative (SFARI) has curated zebrafish lines with mutations in zebrafish ASD risk genes (https://www.sfari.org/resource/zebrafish-models/, accessed on 10 May 2022). To date, eight lines with validated loss-of-function are available for distribution (*arid1b*, *chd8*, *dync1h1*, *fmr1*, *mecp2*, *mef2c*, *pten* and *scn1a/scn2a*). These loss-of-function lines were validated by direct measurement of target mRNA or protein levels [295]. Of note, six genes with loss of function (*cntnap2*, *dyrk1a*, *grin2b*, *nrxn1*, *shank3*, and *syngap1*) have been validated but are not yet available at the Zebrafish International Research Center (ZIRC). Furthermore, based on the association of each gene with autism incidence, SFARI has established a gene scoring system classifying ASD genes into one “syndromic” category or one of three “idiopathic” categories: category 1 (high confidence), 2 (strong candidate), or 3 (suggestive evidence) (https://gene.sfari.org/database/gene-scoring/, accessed on 10 May 2022). Table 9 summarizes these genes with their classification score. Because this review also describes ADHD, when there is an association of the gene with ADHD, we mentioned it.

### 4.2. Pharmacological Zebrafish Models of ASD

#### 4.2.1. Valproic Acid

The most-reported pharmacological model is valproic acid (VPA), a drug that is known to induce autism-like effects in animal models. Originally, VPA was used as an anticonvulsant drug to treat seizures, and studies have demonstrated that embryonic exposure to VPA can lead to ASD in children [296,297,298]. VPA has been implicated in ASD by using various animal models, such as rats, mice and prairie voles [205,299]. Exposure of larval zebrafish to VPA results in phenotypic changes such as reduction of neural cell proliferation in the telencephalon [300] and decreased locomotor activity [301].

#### 4.2.2. Other Drugs

The noncompetitive glutamate N-methyl-D-aspartate (NMDA) receptor antagonist dizocilpine (MK-801) induces impaired shoaling and aggression in zebrafish [302,303]. The abnormal behaviours are reversed by oxytocin and carbetocin but not by the oxytocin receptor antagonist L-368,899 [302]. Acute nicotine administration decreases shoaling behaviour (low effect on shoaling polarization), whereas acute ethanol mildly decreases shoal cohesion and affects polarization [304]. Additionally, the D1 receptor antagonist SCH23390 was demonstrated to decrease social preference in the zebrafish AB strain [305]; however, there was no sign of altered motor function or vision. Lead pollutants are also used to generate ASD zebrafish models, as they decrease shoal cohesion and increase anxiety-like freezing and edge preference [306]. The water-soluble fraction of crude oil also significantly increases anxiety and locomotor activity, decreases repetitive behaviour, and reduces the level of serotonin in zebrafish larvae [306]. Developmental exposure to the organophosphate chlorpyrifos is responsible for a decrease in dopamine levels in zebrafish until adulthood [307].

### 4.3. Genetic Zebrafish Models of ADHD

Several genetic models have been developed to study ADHD in larval and adult zebrafish. *Latrophilin 3 (lphn3)*, a G-protein-coupled receptor belonging to the adhesion subfamily, is a regulator of synaptic function and maintenance in brain regions that mediate locomotor activity, attention, and memory for location and path [308]. Down-regulation of the *lphn3* gene ortholog *lphn3.1* induces hyperactivity and impulsivity in zebrafish, as observed in ADHD patients [75]. The *neuromedin U* (*nmu*) gene plays a role in pain, stress, immune-mediated inflammatory diseases and feeding regulation, and its mutants present a hypoactive phenotype [309]. In addition, KO of the *CLOCK* gene *period1b* (*per1b*) implicated in the circadian rhythm, is responsible for hyperactivity, impulsivity, and circadian disturbance phenotypes similar to ADHD [310]. In the same way, as observed for ASD, *cntnap2* mutants display ADHD-like phenotypes such as hyperactivity and GABAergic deficits in the forebrain [276]. Knockdown of the gene *DEP domain containing 5*, *GATOR1 subcomplex subunit* (*depdc5*), a protein-coding gene, generates mutants that demonstrate hyperactivity and cognitive deficits [311]. Similarly, knocking down the gene *micall2b* ortholog in zebrafish leads to animals with hyperactive/impulsive behaviours. These behaviours were corrected by the administration of atomexine [312]. Furthermore, morpholino knockdown of *exosome component 3* (*exosc3*) in zebrafish embryos causes abnormalities such as microcephaly and poor motility [313]. Moreover, *cyclin K* *(ccnk)* knockdown impairs brain and spinal cord development [314]. The X-linked genetic syndrome associated with mutations in the *taf1* gene causes developmental delay [315]. KO of the gene *bromodomain PHD finger transcription factor* *(bptf)*, suggested to be implicated in the regulation of transcription, causes smaller head size and craniofacial abnormalities [316]. Mutations in the transcription factor *mecp2* gene are observed in more than 90% of Rett syndrome patients who also show signs of mental retardation. Interestingly, *mecp2* KO zebrafish show reduced swimming activity [317] and deficits in *neurotrophic factor* (*bdnf)* gene expression [318]. The gene *per1b* ortholog KO in zebrafish results in hyperactive juveniles, with increased aggressiveness. This mutant zebrafish also shows a decrease in levels of dopamine in the brain [310]. In the case of the 5-HT system, the 5-HT synthesis enzymes TPH1 and TPH2 [319,320,321] and transporter genes serotonin transporter (SerT)/*solute carrier family 6 member 4* (*slc6a4*) which encodes an integral membrane protein that transports the neurotransmitter serotonin from synaptic spaces into presynaptic neurons, have also been demonstrated to impact ADHD phenotypes [322]. Several dopaminergic system-related genes have been linked to ADHD phenotypes. This is the case for the gene encoding the dopaminergic D4 receptor DRD4, the dopaminergic D5 receptor DRD5, and the dopaminergic transporter (DAT)/*solute carrier family 6 member 3* (*slc6a3*) [323,324].

### 4.4. Pharmacological Zebrafish Models of ADHD

Pharmacological models of ADHD using zebrafish are scarce. Zebrafish exposed to 1% alcohol demonstrated motor hyperactivity. The mechanism involved is relevant to dopaminergic neuron stimulation [325]. Similarly, the associative learning performance of embryonic alcohol-exposed fish is impaired, and a small amount of alcohol reaching the embryo results in lasting cognitive deficits [326]. Additionally, neurodevelopmental exposure of zebrafish to ethanol causes learning and memory deficits [326,327,328,329]. Nicotine is a toxicant leading to ADHD in humans; however, few studies have evaluated the effects of nicotine on ADHD phenotypes [32,330,331,332,333].

Similarly, it has been reported that environmental lead (Pb) exposure can result in neurological alterations leading to ADHD phenotypes. For example, Pb exposure decreases the density of axon tracts in the zebrafish midbrain and forebrain, corresponding to the downregulation of two genes at 14 and 16 hpf (hours post-fertilization), respectively [334]. The effects of exposure to the commercial polychlorinated biphenyl (PCB) mixture Aroclor (A) 1254 on visual avoidance behaviour were evaluated in zebrafish larvae at 7 dpf, showing elevated levels of thigmotaxis and higher levels of freezing relative to controls [335]. Methylphenidate, an ADHD medication, was evaluated in early life exposure experiments, and it resulted in a transient increase in dopamine, norepinephrine and serotonin, together with long-term impairment in the predatory avoidance response and spatial learning [336]. The environmental contaminant perfluorooctane sulfonate (PFOS) accumulates in the brain and has potential neurotoxic effects. It was previously shown to induce behavioural alterations such as increased spontaneous hyperactivity, a phenotype that was corrected by the administration of dexamphetamine in zebrafish larvae [337].

## 5. Conclusions

In conclusion, zebrafish and mice are useful models to investigate neurodevelopmental disorders such as ASD or ADHD. There is an increase in research on these disorders using either mouse or zebrafish experiments. However, the choice of a model to investigate a particular aspect of each disorder demands specific considerations, as we described that both mouse and zebrafish models include advantages and limitations. Studies to understand the genetic and neurochemical mechanisms should be considered, together with research on animal models to investigate the behavioural aspects of the disorders. The most important thing to consider is the impact of the proposed research on the scientific community. As demonstrated in this review, the results obtained from zebrafish, taking into consideration the advantages of this model, can be further extrapolated to mouse models and eventually humans, thus providing extensive information on different aspects of the disorders.

## Figures and Tables

**Table 1:** Frequent and common behaviours in ASD patients. This table shows common behaviours observed in ASD patients depending on each ASD phenotype.

ASD Phenotypes	Behaviours
Social communication/Interaction	Usually having little or inconstant eye contact
	Lack of sharing interest, emotion, or pleasure when performing recreational activities
	Difficulty in responding or being slow to respond to signs for attention
	Especially talkative about a favourite topic
	Displaying facial expressions, movements, and gestures not related to a discussed topic
	Change in tone of voice (can even be poetic or robot-like)
	Problems with understanding other people
	Difficulty adjusting behaviours to social situations
Restrictive/Repetitive behaviours	Repeating certain behaviours or phrases (echolalia)
	Having an unusual and prolonged interest in numbers, details, or specific facts
	Exhibiting particularly focused interests, such as interests in objects in motion
	More or less sensitive than a neurotypical person to sensory input (light, sound, clothing, or temperature)
Aptitudes/Potentials	Can learn things in surprising ways and remember specific details and information for long periods
	Excellence in mathematics, science, music, or art disciplines at school

**Table 2:** ADHD phenotypes and their components. The main symptoms observed in ADHD individuals are presented depending on ADHD phenotypes.

ADHD Phenotypes	Symptoms
Inattention	Overlook or miss details and make careless mistakes in every aspect of life
	Difficulty sustaining attention in conversations, lectures, or lengthy reading
	Distracted when spoken to directly
	Lose focus and get easily side-tracked
	Difficulty in organizing, managing time, and meeting deadlines
	Avoid tasks requiring important mental effort
	Often lose personal objects (pencils, books, keys, wallet, phone)
	Easily forget to perform simple daily tasks (homework, appointment)
Hyperactivity-Impulsivity	Fidget and squirm while seated
	Stand up brusquely in situations when staying seated is expected
	Run, dash around, or climb at inappropriate times
	Incapacity to play or conduct an activity quietly
	Excessive talking and always in motion
	Incapacity to wait one’s turn
	Often interrupt or intrude others
	Very active in conversations and finish other people’s phrases or answer without being asked

**Table 3:** ASD and ADHD characteristics. Differences and similarities between ASD and ADHD are presented. In addition, conditions that resemble ASD or ADHD are included.

	ASD	ADHD
Definition	A range of neurodevelopmental conditions that are accompanied by repetitive behaviours and causes difficulty with social skills, communication, and thinking.	A neurodevelopmental disorder characterised by impulsively and difficulty in concentration, attention, and staying still.
Similarities	Poor social skills Difficulty in making eye contact Deficits in attention Difficulty in managing one’s emotions Speech/language delays Treatments involve medication and behavioural therapy	
Differences	Less frequent	Very common
	Social communication skills are impaired	Executive skills are impaired
	Repetitive body movements and preference for routine	High activity level and impulsivity: always moving, talkative, interrupts others
	Restricted interest	Distractibility
	Difficulty in nonverbal communication (difficulty in understanding facial expressions)	Difficulty in memory, forgetful
Other conditions sharing the same symptomatology	Speech delays, hearing problems, or other developmental delays Restricted interests Hyperlexia Psychological disorders such as obsessive compulsive disorder, avoidance personality disorder Lead poisoning Genetic disorders such as tardive dyskinesia, Angelman syndrome, Rett syndrome	Mood disorders such as depression and anxiety Alcohol and substance abuse Dyslexia Conduct and oppositional defiant disorder Bipolar disorder Seizure and sleep disorders Tourette’s syndrome

**Table 4:** Comparison of mouse and zebrafish as animal models in neuroscience. This table shows specific differences between mice (mammalian) and zebrafish (teleost).

	Mouse (*Mus musculus*)	Zebrafish (*Danio rerio*)
Graphical representation	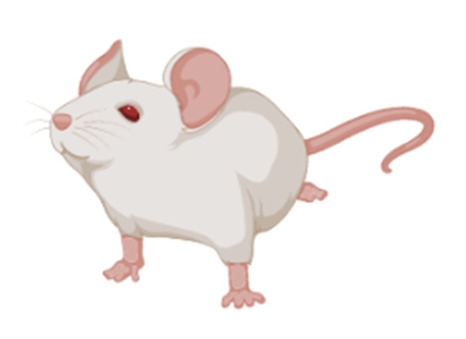	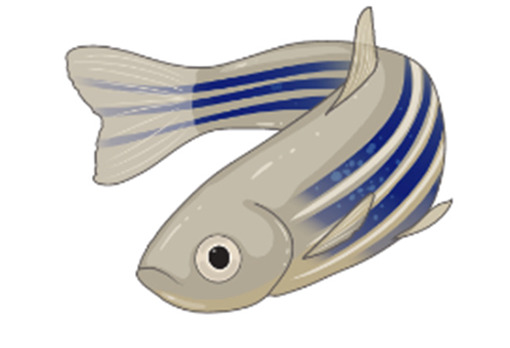
Lifespan	1–2 years	2–5 years
Habitat	Diverse environments	Freshwater streams and rivers
Sexual maturity	Male: 8 weeks; Female: 6 weeks	10–12 weeks (juveniles are hermaphroditic)
Gestation	19–21 days (6–8 pups, 5–10 times/year)	Less than 24 h (200–300 eggs/week)
Advantages in neuroscience research	Can be used to investigate complex behaviours High genetic similarity to humans	Can be used to investigate complex behaviours Excellent and rapid reproductive rate Ease of neural analysis due to their transparent body in early life High genetic similarity to humans
Limitations in neuroscience research	Expensive to maintain Ethical limitations Long experimental cycle	Average flexibility, predictivity and translational value

**Table 7:** Main chemicals used to induce ASD in mouse models. Chemicals are used to induce ASD behaviours in mice.

Drugs	Behaviours Observed	References
Arsenic	Poor sociability and poor social novelty preference	[209]
Bisphenol A	Altered female exploratory and anxiety behaviour, increased levels of affiliation to female stimulus mice and decreased levels of affiliation to male stimulus mice	[210]
Chlorpyrifos	Reduced preference towards an unfamiliar conspecific in the social preference test and reduced social conditioned place preference	[211]
*d*-Amphetamine	Reduction in sociability with no stimulation of locomotor activity	[212]
GABA-A	Reduction in sociability	[212]
Ketamine	Social deficits	[213]
Phencyclidine (PCP)	Reduction in sociability	[214]
Valproic acid	Decreased social interaction, increased repetitive behaviours, lower sensitivity to pain, increased anxiety, reduced locomotor activity Increased fear memories	[206,207]

**Table 8:** Principal zebrafish ASD models and phenotypes observed. This table presents the main zebrafish models in the literature and the reported phenotypes. ENU: ethylnitrosourea; MO: morpholino, TALEN: transcription activator-like effector nucleases; ZFN: zinc-finger nucleases.

Genes	Modification Technique	Phenotypes	References
*AT-rich interaction domain 1B* (*arid1b*)	MO	Reduction in body length and alteration of chondrogenic/osteogenic genes expression	[268]
*Aristaless related homeobox* (*arx*)	MO	Alterations in neurons and brain development	[269]
*Activator of transcription and developmental regulator AUTS2* (*auts2*)	MO	Microcephaly, small head and body zebrafish, reduced locomotor activity	[270]
*Calcium voltage-gated channel subunit alpha1 C* (*cacna1c*)	MO	Cardiac alterations	[271]
*Centrosomal protein 41* (*cep41*)	MO	Neuronal defects and deficits in social behaviour	[272]
*Chromodomain helicase DNA binding protein 2* (*chd2*)	MO	Microcephaly, abnormalities in body shape and motor impairments	[273]
*Chromodomain helicase DNA binding protein 8* (*chd8*)	CRISPR/Cas9, MO	Macrocephaly, decreased gastro-intestinal motility	[274,275]
*Contactin associated protein 2* (*cntnap2*)	ZFN	Decreased forebrain GABAergic neurons at 4 dpf, microcephaly and motor impairments	[276]
*Catenin delta 2* (*ctnnd2*)	MO	Reduced body length and various notochord alterations	[277]
*Dual specificity tyrosine phosphorylation regulated kinase 1A* (*dyrk1a*)	TALEN	Increased brain apoptosis, microcephaly, decreased anxiety and decreased freezing times, deficits in social behaviours	[278]
*Potassium inwardly rectifying channel subfamily J member 10* (*kcnj10*)	MO	Motor and developmental alterations	[279]
*Lysine demethylase 6A* (*kdm6a*)	MO	Reduction in body length and notochord alterations	[280,281]
*Methyl-CpG binding protein 2* (mecp2)	ENU, MO	Neuronal and immune response alterations	[282,283]
*MET proto-oncogene, receptor tyrosine kinase* (*met*)	MO	Increased mortality and neuronal defects	[284]
*Myelin transcription factor 1 like* (*myt1l*)	MO	Loss of oxytocin expression in the preoptic neuroendocrine area	[285]
*Neurobeachin* (*nbea*)	ENU, TALEN	Abnormal response to startle stimuli	[286]
*Nuclear receptor subfamily 3 group C member 2* (*nr3c2*)	CRISPR/Cas9	Alteration in social behaviour and sleep	[287]
*Oxytocin/neurophysin I prepropeptide* (*oxt*)	TALEN	Altered oxytocin signalling and memory alterations	[288]
*Reelin* (*reln*)	TALEN	Altered social behaviour and disrupted serotonin signalling pathway	[289]
*Arginine-glutamic acid dipeptide repeats* (*rere*)	ENU	Deficits in vision and hearing, altered startle response to stimuli	[290]
*SH3 and multiple ankyrin repeat domains 3* (*shank*3)	CRISPR/Cas9, MO	Abnormal mid-hindbrain boundary, increased apoptosis in CNS, decreased GABAergic neurons, impaired social preference, hypoactivity, seizure-like behaviours	[291,292]
*Synaptic Ras GTPase activating protein 1* (*syngap1*)	MO	Microcephaly, developmental delay, high mortality, increased apoptosis in CNS, motor impairment	[293]

**Table 9:** Classification of some ASD genes according to SFARI. ASD gene classification and their ADHD association are presented.

Genes	Names	SFARI Gene Score	ADHD Association
*arid1b*	*AT rich interactive domain 1B*	High confidence, Syndromic (1S)	Yes
*chd8*	*Chromatin helicase DNA-binding protein 8*	High confidence, Syndromic (1S)	Yes
*dync1h1*	*Dynein cytoplasmic 1 heavy chain 1*	High confidence (1)	No
*fmr1*	*Fragile X syndrome mental retardation 1*	High confidence, Syndromic (1S)	Yes
*mecp2*	*Methyl CpG binding protein 2*	High confidence, Syndromic (1S)	Yes
*mef2c*	*Myocyte enhancer factor 2c*	High confidence, Syndromic (1S)	Yes
*pten*	*Phosphatase and tensin homolog*	High confidence, Syndromic (1S)	Yes
*scn1a/scn2a*	*Sodium channel voltage-gated, type I-like, alpha subunit/type II-like, alpha subunit*	High confidence, Syndromic (1S)	Yes
*cntnap2*	*Contactin associated protein-like 2*	Syndromic (S)	Yes
*dyrk1a*	*Dual-specificity tyrosine-(Y)-phosphorylation regulated kinase 1A*	High confidence, Syndromic (1S)	No
*grin2b*	*Glutamate receptor ionotropic N-methyl D-aspartate 2B*	High confidence (1)	Yes
*nrxn1*	*Neurexin 1*	High confidence (1)	Yes
*shank3*	*SH3 and multiple ankyrin repeat domains*	High confidence, Syndromic (1S)	No
*syngap1*	*Synaptic Ras GTPase activating protein 1*	High confidence, Syndromic (1S)	Yes

## Data Availability

Data and tools described in this manuscript are available upon reasonable request.

## References

[1] Fernandes J. (2014). DSM Cautionary Statement for Forensic Use of DSM-5. Diagnostic and Statistical Manual of Mental Disorders.

[2] CDC Autism Prevalence Studies|Data|Centers for Disease Control and Prevention. https://data.cdc.gov/Public-Health-Surveillance/autism-prevalence-studies/9mw4-6adp.

[3] Lyall K., Croen L., Daniels J., Fallin M.D., Ladd-Acosta C., Lee B.K., Park B.Y., Snyder N.W., Schendel D., Volk H. (2017). The Changing Epidemiology of Autism Spectrum Disorders. Annu. Rev. Public Health.

[4] (2017). IHME Prevalence of Autistic Spectrum Disorder. https://ourworldindata.org/grapher/prevalence-of-autistic-spectrum.

[5] IHME GBD Results Tool|GHDx. https://ghdx.healthdata.org/gbd-results-tool.

[6] NIMH Autism Spectrum Disorder. https://www.nimh.nih.gov/health/topics/autism-spectrum-disorders-asd.

[7] Autism Speaks (2017). Autism and Health: Advances in Understanding and Treating the Health Conditions That Frequently Accompany Autism.

[8] Colvert E., Tick B., McEwen F., Stewart C., Curran S.R., Woodhouse E., Gillan N., Hallett V., Lietz S., Garnett T. (2015). Heritability of Autism Spectrum Disorder in a UK Population-Based Twin Sample. JAMA Psychiatry.

[9] Durkin M.S., Maenner M.J., Newschaffer C.J., Lee L.C., Cunniff C.M., Daniels J.L., Kirby R.S., Leavitt L., Miller L., Zahorodny W. (2008). Advanced Parental Age and the Risk of Autism Spectrum Disorder. Am. J. Epidemiol..

[10] Hallmayer J., Cleveland S., Torres A., Phillips J., Cohen B., Torigoe T., Miller J., Fedele A., Collins J., Smith K. (2011). Genetic Heritability and Shared Environmental Factors Among Twin Pairs with Autism. Arch. Gen. Psychiatry.

[11] Ronald A., Happé F., Bolton P., Butcher L.M., Price T.S., Wheelwright S., Baron-Cohen S., Plomin R. (2006). Genetic Heterogeneity between the Three Components of the Autism Spectrum: A Twin Study. J. Am. Acad. Child Adolesc. Psychiatry.

[12] Ozonoff S., Young G.S., Carter A., Messinger D., Yirmiya N., Zwaigenbaum L., Bryson S., Carver L.J., Constantino J.N., Dobkins K. (2011). Recurrence Risk for Autism Spectrum Disorders: A Baby Siblings Research Consortium Study. Pediatrics.

[13] Sumi S., Taniai H., Miyachi T., Tanemura M. (2006). Sibling Risk of Pervasive Developmental Disorder Estimated by Means of an Epidemiologic Survey in Nagoya, Japan. J. Hum. Genet..

[14] Rosenberg R.E., Law J.K., Yenokyan G., McGready J., Kaufmann W.E., Law P.A. (2009). Characteristics and Concordance of Autism Spectrum Disorders among 277 Twin Pairs. Arch. Pediatr. Adolesc. Med..

[15] Taniai H., Nishiyama T., Miyachi T., Imaeda M., Sumi S. (2008). Genetic Influences on the Broad Spectrum of Autism: Study of Proband-Ascertained Twins. Am. J. Med. Genet. B. Neuropsychiatr. Genet..

[16] Moss J., Chris O. (2012). Autism in Genetic Syndromes: Implications for Assessment and Intervention.

[17] Garvía Peñuelas B. (2017). Down Syndrome and Autism Spectrum Disorder. Int. Med. Rev. Down Syndr..

[18] Hagerman R., Hoem G., Hagerman P. (2010). Fragile X and Autism: Intertwined at the Molecular Level Leading to Targeted Treatments. Mol. Autism.

[19] Jain A., Marshall J., Buikema A., Bancroft T., Kelly J.P., Newschaffer C.J. (2015). Autism Occurrence by MMR Vaccine Status among US Children with Older Siblings with and without Autism. JAMA.

[20] Uno Y., Uchiyama T., Kurosawa M., Aleksic B., Ozaki N. (2015). Early Exposure to the Combined Measles-Mumps-Rubella Vaccine and Thimerosal-Containing Vaccines and Risk of Autism Spectrum Disorder. Vaccine.

[21] Gadad B.S., Li W., Yazdani U., Grady S., Johnson T., Hammond J., Gunn H., Curtis B., English C., Yutuc V. (2015). Administration of Thimerosal-Containing Vaccines to Infant Rhesus Macaques Does Not Result in Autism-like Behavior or Neuropathology. Proc. Natl. Acad. Sci. USA.

[22] Zerbo O., Qian Y., Yoshida C., Fireman B.H., Klein N.P., Croen L.A. (2017). Association Between Influenza Infection and Vaccination during Pregnancy and Risk of Autism Spectrum Disorder. JAMA Pediatr..

[23] CDC What Is Autism Spectrum Disorder?. https://www.cdc.gov/ncbddd/autism/facts.html.

[24] CDC Diagnostic Criteria|Autism Spectrum Disorder (ASD)|NCBDDD|CDC. https://www.cdc.gov/ncbddd/autism/hcp-dsm.html.

[25] WebMD Autism Therapies. https://www.webmd.com/brain/autism/therapies-to-help-with-autism.

[26] Cooper K., Loades M.E., Russell A. (2018). Adapting Psychological Therapies for Autism—Therapist Experience, Skills and Confidence. Res. Autism Spectr. Disord..

[27] AltogetherAutism Cognitive Behavioural Therapy Modifications for Those on the Autism Spectrum—Altogether Autism. https://www.altogetherautism.org.nz/cognitive-behavioural-therapy-modifications-for-those-on-the-autism-spectrum/.

[28] Spain D., Sin J., Chalder T., Murphy D., Happé F. (2015). Cognitive Behaviour Therapy for Adults with Autism Spectrum Disorders and Psychiatric Co-Morbidity: A Review. Res. Autism Spectr. Disord..

[29] Fayyad J., Sampson N.A., Hwang I., Adamowski T., Aguilar-Gaxiola S., Al-Hamzawi A., Andrade L.H.S.G., Borges G., de Girolamo G., Florescu S. (2017). The Descriptive Epidemiology of DSM-IV Adult ADHD in the World Health Organization World Mental Health Surveys. Atten. Defic. Hyperact. Disord..

[30] Ebejer J.L., Medland S.E., van der Werf J., Gondro C., Henders A.K., Lynskey M., Martin N.G., Duffy D.L. (2012). Attention Deficit Hyperactivity Disorder in Australian Adults: Prevalence, Persistence, Conduct Problems and Disadvantage. PLoS ONE.

[31] Volkow N.D., Swanson J.M. (2013). Adult Attention Deficit–Hyperactivity Disorder. N. Engl. J. Med..

[32] McClernon F.J., Kollins S.H. (2008). ADHD and Smoking. Ann. N. Y. Acad. Sci..

[33] Jung Y., Hsieh L.S., Lee A.M., Zhou Z., Coman D., Heath C.J., Hyder F., Mineur Y.S., Yuan Q., Goldman D. (2016). An Epigenetic Mechanism Mediates Developmental Nicotine Effects on Neuronal Structure and Behavior. Nat. Neurosci..

[34] Rahmani Z., Fayyazi Bordbar M.R., Dibaj M., Alimardani M., Moghbeli M. (2021). Genetic and Molecular Biology of Autism Spectrum Disorder among Middle East Population: A Review. Hum. Genom..

[35] Noroozi R., Taheri M., Movafagh A., Mirfakhraie R., Solgi G., Sayad A., Mazdeh M., Darvish H. (2016). Glutamate Receptor, Metabotropic 7 (*GRM7*) Gene Variations and Susceptibility to Autism: A Case–Control Study. Autism Res..

[36] Yang Y., Pan C. (2013). Role of Metabotropic Glutamate Receptor 7 in Autism Spectrum Disorders: A Pilot Study. Life Sci..

[37] Hosseinpour M., Mashayekhi F., Bidabadi E., Salehi Z. (2017). Neuropilin-2 Rs849563 Gene Variations and Susceptibility to Autism in Iranian Population: A Case-Control Study. Metab. Brain Dis..

[38] Giulivi C., Zhang Y.F., Omanska-Klusek A., Ross-Inta C., Wong S., Hertz-Picciotto I., Tassone F., Pessah I.N. (2010). Mitochondrial Dysfunction in Autism. JAMA.

[39] Idriss H.T., Naismith J.H. (2000). TNFα and the TNF Receptor Superfamily: Structure-Function Relationship(s). Microsc. Res. Technol..

[40] Eftekharian M.M., Ghafouri-Fard S., Noroozi R., Omrani M.D., Arsang-jang S., Ganji M., Gharzi V., Noroozi H., Komaki A., Mazdeh M. (2018). Cytokine Profile in Autistic Patients. Cytokine.

[41] Vaht M., Kiive E., Veidebaum T., Harro J. (2016). A Functional Vesicular Monoamine Transporter 1 (*VMAT1*) Gene Variant Is Associated with Affect and the Prevalence of Anxiety, Affective, and Alcohol Use Disorders in a Longitudinal Population-Representative Birth Cohort Study. Int. J. Neuropsychopharmacol..

[42] Uitterlinden A.G., Fang Y., Van Meurs J.B.J., Pols H.A.P., Van Leeuwen J.P.T.M. (2004). Genetics and Biology of Vitamin D Receptor Polymorphisms. Gene.

[43] Emamalizadeh B., Jamshidi J., Movafagh A., Ohadi M., Khaniani M.S., Kazeminasab S., Biglarian A., Taghavi S., Motallebi M., Fazeli A. (2016). RIT2 Polymorphisms: Is There a Differential Association?. Mol. Neurobiol..

[44] Mihai Bădescu G., Fîlfan M., Sandu R.E., Surugiu R., Ciobanu O., Popa-Wagner A. (2016). Molecular Mechanisms Underlying Neurodevelopmental Disorders, ADHD and Autism. Rom. J. Morphol. Embryol..

[45] Hoogman M., Onnink M., Cools R., Aarts E., Kan C., Arias Vasquez A., Buitelaar J., Franke B. (2013). The Dopamine Transporter Haplotype and Reward-Related Striatal Responses in Adult ADHD. Eur. Neuropsychopharmacol..

[46] Binkovitz L., Thacker P. (2015). What Does Molecular Imaging Reveal about the Causes of ADHD and the Potential for Better Management?. Curr. Psychiatr..

[47] Volkow N.D., Wang G.J., Fowler J.S., Gatley S.J., Logan J., Ding Y.S., Hitzemann R., Pappas N. (1998). Dopamine Transporter Occupancies in the Human Brain Induced by Therapeutic Doses of Oral Methylphenidate. Am. J. Psychiatry.

[48] Li J.J., Lee S.S. (2013). Interaction of Dopamine Transporter Gene and Observed Parenting Behaviors on Attention-Deficit/Hyperactivity Disorder: A Structural Equation Modeling Approach. J. Clin. Child Adolesc. Psychol..

[49] Volkow N.D., Wang G.-J., Kollins S.H., Wigal T.L., Newcorn J.H., Telang F., Fowler J.S., Zhu W., Logan J., Ma Y. (2009). Evaluating Dopamine Reward Pathway in ADHD. JAMA.

[50] Lou H.C., Rosa P., Pryds O., Karrebæk H., Lunding J., Cumming P., Gjedde A. (2004). ADHD: Increased Dopamine Receptor Availability Linked to Attention Deficit and Low Neonatal Cerebral Blood Flow. Dev. Med. Child Neurol..

[51] Baird A.L., Coogan A.N., Siddiqui A., Donev R.M., Thome J. (2012). Adult Attention-Deficit Hyperactivity Disorder Is Associated with Alterations in Circadian Rhythms at the Behavioural, Endocrine and Molecular Levels. Mol. Psychiatry.

[52] Ji E.-S., Kim C.-J., Park J.H., Bahn G.H. (2014). Duration-Dependence of the Effect of Treadmill Exercise on Hyperactivity in Attention Deficit Hyperactivity Disorder Rats. J. Exerc. Rehabil..

[53] Dibner C., Schibler U., Albrecht U. (2010). The Mammalian Circadian Timing System: Organization and Coordination of Central and Peripheral Clocks. Annu. Rev. Physiol..

[54] Cryan J.F., Holmes A. (2005). Model Organisms: The Ascent of Mouse: Advances in Modelling Human Depression and Anxiety. Nat. Rev. Drug Discov..

[55] Choi S.H., Bylykbashi E., Chatila Z.K., Lee S.W., Pulli B., Clemenson G.D., Kim E., Rompala A., Oram M.K., Asselin C. (2018). Induced Adult Neurogenesis plus BDNF Mimicks the Effects of Exercise on Cognition in an Alzheimer’s Mouse Model. Science.

[56] Kodera K., Matsui H. (2022). Zebrafish, Medaka and Turquoise Killifish for Understanding Human Neurodegenerative/Neurodevelopmental Disorders. Int. J. Mol. Sci..

[57] Matsui H., Sugie A. (2017). An Optimized Method for Counting Dopaminergic Neurons in Zebrafish. PLoS ONE.

[58] Gerlai R. (2019). Reproducibility and Replicability in Zebrafish Behavioral Neuroscience Research. Pharmacol. Biochem. Behav..

[59] Fabian P., Tseng K.C., Smeeton J., Lancman J.J., Duc Si Dong P., Cerny R., Gage Crump J. (2020). Lineage Analysis Reveals an Endodermal Contribution to the Vertebrate Pituitary. Science.

[60] Wang W., Hu C.-K., Zeng A., Alegre D., Hu D., Gotting K., Ortega Granillo A., Wang Y., Robb S., Schnittker R. (2020). Changes in Regeneration-Responsive Enhancers Shape Regenerative Capacities in Vertebrates. Science.

[61] Pensado-López A., Veiga-Rúa S., Carracedo Á., Allegue C., Sánchez L. (2020). Experimental Models to Study Autism Spectrum Disorders: HiPSCs, Rodents and Zebrafish. Genes.

[62] Wöhr M., Scattoni M.L. (2013). Behavioural Methods Used in Rodent Models of Autism Spectrum Disorders: Current Standards and New Developments. Behav. Brain Res..

[63] Silverman J.L., Yang M., Lord C., Crawley J.N. (2010). Behavioural Phenotyping Assays for Mouse Models of Autism. Nat. Rev. Neurosci..

[64] Davids E., Zhang K., Tarazi F.I., Baldessarini R.J. (2003). Animal Models of Attention-Deficit Hyperactivity Disorder. Brain Res. Rev..

[65] Mortimer N., Ganster T., O’Leary A., Popp S., Freudenberg F., Reif A., Soler Artigas M., Ribasés M., Ramos-Quiroga J.A., Lesch K.P. (2019). Dissociation of Impulsivity and Aggression in Mice Deficient for the ADHD Risk Gene Adgrl3: Evidence for Dopamine Transporter Dysregulation. Neuropharmacology.

[66] Jhun M., Panwar A., Cordner R., Irvin D.K., Veiga L., Yeager N., Pechnick R.N., Schubloom H., Black K.L., Wheeler C.J. (2020). CD103 Deficiency Promotes Autism (ASD) and Attention-Deficit Hyperactivity Disorder (ADHD) Behavioral Spectra and Reduces Age-Related Cognitive Decline. Front. Neurol..

[67] Moy S.S., Nadler J.J., Perez A., Barbaro R.P., Johns J.M., Magnuson T.R., Piven J., Crawley J.N. (2004). Sociability and Preference for Social Novelty in Five Inbred Strains: An Approach to Assess Autistic-like Behavior in Mice. Genes Brain Behav..

[68] McFarlane H.G., Kusek G.K., Yang M., Phoenix J.L., Bolivar V.J., Crawley J.N. (2008). Autism-like Behavioral Phenotypes in BTBR T+tf/J Mice. Genes Brain Behav..

[69] Moretz J.A., Martins E.P., Robison B.D. (2007). The Effects of Early and Adult Social Environment on Zebrafish (*Danio rerio*) Behavior. Environ. Biol. Fishes.

[70] Gerlai R., Lahav M., Guo S., Rosenthal A. (2000). Drinks like a Fish: Zebra Fish (*Danio rerio*) as a Behavior Genetic Model to Study Alcohol Effects. Pharmacol. Biochem. Behav..

[71] Pearson B.L., Pobbe R.L.H., Defensor E.B., Oasay L., Bolivar V.J., Blanchard D.C., Blanchard R.J. (2011). Motor and Cognitive Stereotypies in the BTBR T+tf/J Mouse Model of Autism. Genes Brain Behav..

[72] Reynolds S., Urruela M., Devine D.P. (2013). Effects of Environmental Enrichment on Repetitive Behaviors in the BTBR T+tf/J Mouse Model of Autism. Autism Res..

[73] MacPhail R.C., Brooks J., Hunter D.L., Padnos B., Irons T.D., Padilla S. (2009). Locomotion in Larval Zebrafish: Influence of Time of Day, Lighting and Ethanol. Neurotoxicology.

[74] Lange M., Norton W., Coolen M., Chaminade M., Merker S., Proft F., Schmitt A., Vernier P., Lesch K.-P., Bally-Cuif L. (2012). The ADHD-Susceptibility Gene Lphn3.1 Modulates Dopaminergic Neuron Formation and Locomotor Activity during Zebrafish Development. Mol. Psychiatry.

[75] Ingebretson J.J., Masino M.A. (2013). Quantification of Locomotor Activity in Larval Zebrafish: Considerations for the Design of High-Throughput Behavioral Studies. Front. Neural Circuits.

[76] Ulhaq M., Örn S., Carlsson G., Morrison D.A., Norrgren L. (2013). Locomotor Behavior in Zebrafish (*Danio rerio*) Larvae Exposed to Perfluoroalkyl Acids. Aquat. Toxicol..

[77] Grossman L., Utterback E., Stewart A., Gaikwad S., Chung K.M., Suciu C., Wong K., Elegante M., Elkhayat S., Tan J. (2010). Characterization of Behavioral and Endocrine Effects of LSD on Zebrafish. Behav. Brain Res..

[78] Spinello C., Yang Y., Macrì S., Porfiri M. (2019). Zebrafish Adjust Their Behavior in Response to an Interactive Robotic Predator. Front. Robot. AI.

[79] Amaral V.C.S., Santos Gomes K., Nunes-de-Souza R.L. (2010). Increased Corticosterone Levels in Mice Subjected to the Rat Exposure Test. Horm. Behav..

[80] Roullet F.I., Wöhr M., Crawley J.N. (2011). Female Urine-Induced Male Mice Ultrasonic Vocalizations, but Not Scent-Marking, Is Modulated by Social Experience. Behav. Brain Res..

[81] Wöhr M., Roullet F.I., Crawley J.N. (2011). Reduced Scent Marking and Ultrasonic Vocalizations in the BTBR T+tf/J Mouse Model of Autism. Genes Brain Behav..

[82] Parker M.O., Brock A.J., Sudwarts A., Brennan C.H. (2014). Atomoxetine Reduces Anticipatory Responding in a 5-Choice Serial Reaction Time Task for Adult Zebrafish. Psychopharmacology.

[83] Parker M.O., Ife D., Ma J., Pancholi M., Smeraldi F., Straw C., Brennan C.H. (2013). Development and Automation of a Test of Impulse Control in Zebrafish. Front. Syst. Neurosci..

[84] Wiprich M.T., Zanandrea R., Altenhofen S., Bonan C.D. (2020). Influence of 3-Nitropropionic Acid on Physiological and Behavioral Responses in Zebrafish Larvae and Adults. Comp. Biochem. Physiol. Part C Toxicol. Pharmacol..

[85] Blaser R.E., Rosemberg D.B. (2012). Measures of Anxiety in Zebrafish (*Danio rerio*): Dissociation of Black/White Preference and Novel Tank Test. PLoS ONE.

[86] Egan R.J., Bergner C.L., Hart P.C., Cachat J.M., Canavello P.R., Elegante M.F., Elkhayat S.I., Bartels B.K., Tien A.K., Tien D.H. (2009). Understanding Behavioral and Physiological Phenotypes of Stress and Anxiety in Zebrafish. Behav. Brain Res..

[87] Maximino C., Lima M.G., de Jesus Oliveira Batista E., Oliveira K.R.H.M., Herculano A.M. (2015). Interaction between 5-HT1B Receptors and Nitric Oxide in Zebrafish Responses to Novelty. Neurosci. Lett..

[88] Jager A., Kanters D., Geers F., Buitelaar J.K., Kozicz T., Glennon J.C. (2019). Methylphenidate Dose-Dependently Affects Aggression and Improves Fear Extinction and Anxiety in BALB/CJ Mice. Front. Psychiatry.

[89] Koolhaas J.M., Coppens C.M., de Boer S.F., Buwalda B., Meerlo P., Timmermans P.J.A. (2013). The Resident-Intruder Paradigm: A Standardized Test for Aggression, Violence and Social Stress. J. Vis. Exp..

[90] Zimmermann F.F., Gaspary K.V., Leite C.E., De Paula Cognato G., Bonan C.D. (2015). Embryological Exposure to Valproic Acid Induces Social Interaction Deficits in Zebrafish (*Danio rerio*): A Developmental Behavior Analysis. Neurotoxicol. Teratol..

[91] Buske C., Gerlai R. (2011). Shoaling Develops with Age in Zebrafish (*Danio rerio*). Prog. Neuro-Psychopharmacol. Biol. Psychiatry.

[92] Dougnon G., Ito M. (2020). Sedative Effects of the Essential Oil from the Leaves of *Lantana Camara* Occurring in the Republic of Benin via Inhalation in Mice. J. Nat. Med..

[93] Dougnon G., Ito M. (2021). Essential Oil from the Leaves of *Chromolaena Odorata*, and Sesquiterpene Caryophyllene Oxide Induce Sedative Activity in Mice. Pharmaceuticals.

[94] Dougnon G., Ito M. (2021). Role of Ascaridole and P-Cymene in the Sleep-Promoting Effects of *Dysphania Ambrosioides* Essential Oil via the GABAergic System in a DdY Mouse Inhalation Model. J. Nat. Prod..

[95] Baker K.B., Wray S.P., Ritter R., Mason S., Lanthorn T.H., Savelieva K.V. (2010). Male and Female Fmr1 Knockout Mice on C57 Albino Background Exhibit Spatial Learning and Memory Impairments. Genes Brain. Behav..

[96] Magara F., Ricceri L., Wolfer D.P., Lipp H.P. (2000). The Acallosal Mouse Strain I/LnJ: A Putative Model of ADHD?. Neurosci. Biobehav. Rev..

[97] Abrahams B.S., Arking D.E., Campbell D.B., Mefford H.C., Morrow E.M., Weiss L.A., Menashe I., Wadkins T., Banerjee-Basu S., Packer A. (2013). SFARI Gene 2.0: A Community-Driven Knowledgebase for the Autism Spectrum Disorders (ASDs). Mol. Autism.

[98] Meyza K.Z., Defensor E.B., Jensen A.L., Corley M.J., Pearson B.L., Pobbe R.L.H., Bolivar V.J., Blanchard D.C., Blanchard R.J. (2013). The BTBR T+tf/J Mouse Model for Autism Spectrum Disorders–in Search of Biomarkers. Behav. Brain Res..

[99] Scattoni M.L., Gandhy S.U., Ricceri L., Crawley J.N. (2008). Unusual Repertoire of Vocalizations in the BTBR T+tf/J Mouse Model of Autism. PLoS ONE.

[100] UniProt Find Your Protein. https://www.uniprot.org/.

[101] Bozdagi O., Sakurai T., Papapetrou D., Wang X., Dickstein D.L., Takahashi N., Kajiwara Y., Yang M., Katz A.M., Scattoni M. (2010). Haploinsufficiency of the Autism-Associated Shank3 Gene Leads to Deficits in Synaptic Function, Social Interaction, and Social Communication. Mol. Autism.

[102] Peça J., Feliciano C., Ting J.T., Wang W., Wells M.F., Venkatraman T.N., Lascola C.D., Fu Z., Feng G. (2011). Shank3 Mutant Mice Display Autistic-like Behaviours and Striatal Dysfunction. Nature.

[103] Crawley J.N. (2012). Translational Animal Models of Autism and Neurodevelopmental Disorders. Dialogues Clin. Neurosci..

[104] Pieretti M., Zhang F., Fu Y.H., Warren S.T., Oostra B.A., Caskey C.T., Nelson D.L. (1991). Absence of Expression of the FMR-1 Gene in Fragile X Syndrome. Cell.

[105] Fu Y.H., Kuhl D.P.A., Pizzuti A., Pieretti M., Sutcliffe J.S., Richards S., Verkert A.J.M.H., Holden J.J.A., Fenwick R.G., Warren S.T. (1991). Variation of the CGG Repeat at the Fragile X Site Results in Genetic Instability: Resolution of the Sherman Paradox. Cell.

[106] Mineur Y.S., Huynh L.X., Crusio W.E. (2006). Social Behavior Deficits in the Fmr1 Mutant Mouse. Behav. Brain Res..

[107] Chen L.Y., Rex C.S., Babayan A.H., Kramár E.A., Lynch G., Gall C.M., Lauterborn J.C. (2010). Physiological Activation of Synaptic Rac>PAK (p-21 Activated Kinase) Signaling Is Defective in a Mouse Model of Fragile X Syndrome. J. Neurosci..

[108] Spencer C.M., Graham D.F., Yuva-Paylor L.A., Nelson D.L., Paylor R. (2008). Social Behavior in Fmr1 Knockout Mice Carrying a Human FMR1 Transgene. Behav. Neurosci..

[109] Huber K.M., Gallagher S.M., Warren S.T., Bear M.F. (2002). Altered Synaptic Plasticity in a Mouse Model of Fragile X Mental Retardation. Proc. Natl. Acad. Sci. USA.

[110] Dölen G., Osterweil E., Rao B.S.S., Smith G.B., Auerbach B.D., Chattarji S., Bear M.F. (2007). Correction of Fragile X Syndrome in Mice. Neuron.

[111] LaSalle J.M., Reiter L.T., Chamberlain S.J. (2015). Epigenetic Regulation of UBE3A and Roles in Human Neurodevelopmental Disorders. Epigenomics.

[112] Smith S.E.P., Zhou Y.D., Zhang G., Jin Z., Stoppel D.C., Anderson M.P. (2011). Increased Gene Dosage of Ube3a Results in Autism Traits and Decreased Glutamate Synaptic Transmission in Mice. Sci. Transl. Med..

[113] Scott-Van Zeeland A.A., Abrahams B.S., Alvarez-Retuerto A.I., Sonnenblick L.I., Rudie J.D., Ghahremani D., Mumford J.A., Poldrack R.A., Dapretto M., Geschwind D.H. (2010). Altered Functional Connectivity in Frontal Lobe Circuits Is Associated with Variation in the Autism Risk Gene CNTNAP2. Sci. Transl. Med..

[114] Moy S.S., Riddick N.V., Nikolova V.D., Teng B.L., Agster K.L., Nonneman R.J., Young N.B., Baker L.K., Nadler J.J., Bodfish J.W. (2014). Repetitive Behavior Profile and Supersensitivity to Amphetamine in the C58/J Mouse Model of Autism. Behav. Brain Res..

[115] Ergaz Z., Weinstein-Fudim L., Ornoy A. (2016). Genetic and Non-Genetic Animal Models for Autism Spectrum Disorders (ASD). Reprod. Toxicol..

[116] Samaco R.C., Mcgraw C.M., Ward C.S., Sun Y., Neul J.L., Zoghbi H.Y. (2013). Female Mecp2+/− Mice Display Robust Behavioral Deficits on Two Different Genetic Backgrounds Providing a Framework for Pre-Clinical Studies. Hum. Mol. Genet..

[117] Chao H.T., Chen H., Samaco R.C., Xue M., Chahrour M., Yoo J., Neul J.L., Gong S., Lu H.C., Heintz N. (2010). Dysfunction in GABA Signalling Mediates Autism-like Stereotypies and Rett Syndrome Phenotypes. Nature.

[118] Sztainberg Y., Chen H.M., Swann J.W., Hao S., Tang B., Wu Z., Tang J., Wan Y.W., Liu Z., Rigo F. (2015). Reversal of Phenotypes in MECP2 Duplication Mice Using Genetic Rescue or Antisense Oligonucleotides. Nature.

[119] Wenderski W., Wang L., Krokhotin A., Walsh J.J., Li H., Shoji H., Ghosh S., George R.D., Miller E.L., Elias L. (2020). Loss of the Neural-Specific BAF Subunit ACTL6B Relieves Repression of Early Response Genes and Causes Recessive Autism. Proc. Natl. Acad. Sci. USA.

[120] Sragovich S., Malishkevich A., Piontkewitz Y., Giladi E., Touloumi O., Lagoudaki R., Grigoriadis N., Gozes I. (2019). The Autism/Neuroprotection-Linked ADNP/NAP Regulate the Excitatory Glutamatergic Synapse. Transl. Psychiatry.

[121] Vulih-Shultzman I., Pinhasov A., Mandel S., Grigoriadis N., Touloumi O., Pittel Z., Gozes I. (2007). Activity-Dependent Neuroprotective Protein Snippet NAP Reduces Tau Hyperphosphorylation and Enhances Learning in a Novel Transgenic Mouse Model. J. Pharmacol. Exp. Ther..

[122] Amram N., Hacohen-Kleiman G., Sragovich S., Malishkevich A., Katz J., Touloumi O., Lagoudaki R., Grigoriadis N.C., Giladi E., Yeheskel A. (2016). Sexual Divergence in Microtubule Function: The Novel Intranasal Microtubule Targeting SKIP Normalizes Axonal Transport and Enhances Memory. Mol. Psychiatry.

[123] Dere E., Dahm L., Lu D., Hammerschmidt K., Ju A., Tantra M., Kästner A., Chowdhury K., Ehrenreich H. (2014). Heterozygous Ambra1 Deficiency in Mice: A Genetic Trait with Autism-like Behavior Restricted to the Female Gender. Front. Behav. Neurosci..

[124] Carbonell A.U., Cho C.H., Tindi J.O., Counts P.A., Bates J.C., Erdjument-Bromage H., Cvejic S., Iaboni A., Kvint I., Rosensaft J. (2019). Haploinsufficiency in the ANKS1B Gene Encoding AIDA-1 Leads to a Neurodevelopmental Syndrome. Nat. Commun..

[125] Nakamura T., Arima-Yoshida F., Sakaue F., Nasu-Nishimura Y., Takeda Y., Matsuura K., Akshoomoff N., Mattson S.N., Grossfeld P.D., Manabe T. (2016). PX-RICS-Deficient Mice Mimic Autism Spectrum Disorder in Jacobsen Syndrome through Impaired GABAA Receptor Trafficking. Nat. Commun..

[126] Lu D.H., Liao H.M., Chen C.H., Tu H.J., Liou H.C., Gau S.S.F., Fu W.M. (2018). Impairment of Social Behaviors in Arhgef10 Knockout Mice. Mol. Autism.

[127] Celen C., Chuang J.C., Luo X., Nijem N., Walker A.K., Chen F., Zhang S., Chung A.S., Nguyen L.H., Nassour I. (2017). Arid1b Haploinsufficient Mice Reveal Neuropsychiatric Phenotypes and Reversible Causes of Growth Impairment. eLife.

[128] Shibutani M., Horii T., Shoji H., Morita S., Kimura M., Terawaki N., Miyakawa T., Hatada I. (2017). Arid1b Haploinsufficiency Causes Abnormal Brain Gene Expression and Autism-Related Behaviors in Mice. Int. J. Mol. Sci..

[129] Brinkmeier M.L., Geister K.A., Jones M., Waqas M., Maillard I., Camper S.A. (2015). The Histone Methyltransferase Gene Absent, Small, or Homeotic Discs-1 Like Is Required for Normal Hox Gene Expression and Fertility in Mice. Biol. Reprod..

[130] Zhu Τ., Liang C., Li D., Tian M., Liu S., Gao G., Guan J.-S. (2016). Histone Methyltransferase Ash1L Mediates Activity-Dependent Repression of Neurexin-1α. Sci. Rep..

[131] Kerr D.J., Marsillo A., Guariglia S.R., Budylin T., Sadek R., Menkes S., Chauhan A., Wen G.Y., McCloskey D.P., Wieraszko A. (2016). Aberrant Hippocampal Atp8a1 Levels Are Associated with Altered Synaptic Strength, Electrical Activity, and Autistic-like Behavior. Biochim. Biophys. Acta.

[132] Oaks A.W., Zamarbide M., Tambunan D.E., Santini E., Di Costanzo S., Pond H.L., Johnson M.W., Lin J., Gonzalez D.M., Boehler J.F. (2017). Cc2d1a Loss of Function Disrupts Functional and Morphological Development in Forebrain Neurons Leading to Cognitive and Social Deficits. Cereb. Cortex.

[133] Wersinger S.R., Ginns E.I., O’Carroll A.M., Lolait S.J., Young W.S. (2002). Vasopressin V1b Receptor Knockout Reduces Aggressive Behavior in Male Mice. Mol. Psychiatry.

[134] Scattoni M.L., McFarlane H.G., Zhodzishsky V., Caldwell H.K., Young W.S., Ricceri L., Crawley J.N. (2008). Reduced Ultrasonic Vocalizations in Vasopressin 1b Knockout Mice. Behav. Brain Res..

[135] Ohashi R., Takao K., Miyakawa T., Shiina N. (2016). Comprehensive Behavioral Analysis of RNG105 (Caprin1) Heterozygous Mice: Reduced Social Interaction and Attenuated Response to Novelty. Sci. Rep..

[136] Nagarajan P., Onami T.M., Rajagopalan S., Kania S., Donnell R., Venkatachalam S. (2009). Role of Chromodomain Helicase DNA-Binding Protein 2 in DNA Damage Response Signaling and Tumorigenesis. Oncogene.

[137] Kim Y.J., Khoshkhoo S., Frankowski J.C., Zhu B., Abbasi S., Lee S., Wu Y.E., Hunt R.F. (2018). Chd2 Is Necessary for Neural Circuit Development and Long-Term Memory. Neuron.

[138] Durak O., Gao F., Kaeser-Woo Y.J., Rueda R., Martorell A.J., Nott A., Liu C.Y., Watson L.A., Tsai L.H. (2016). Chd8 Mediates Cortical Neurogenesis via Transcriptional Regulation of Cell Cycle and Wnt Signaling. Nat. Neurosci..

[139] Gompers A.L., Su-Feher L., Ellegood J., Copping N.A., Riyadh M.A., Stradleigh T.W., Pride M.C., Schaffler M.D., Wade A.A., Catta-Preta R. (2017). Germline Chd8 Haploinsufficiency Alters Brain Development in Mouse. Nat. Neurosci..

[140] Jung H., Park H., Choi Y., Kang H., Lee E., Kweon H., Roh J.D., Ellegood J., Choi W., Kang J. (2018). Sexually Dimorphic Behavior, Neuronal Activity, and Gene Expression in Chd8-Mutant Mice. Nat. Neurosci..

[141] Miyata S., Taniguchi M., Koyama Y., Shimizu S., Tanaka T., Yasuno F., Yamamoto A., Iida H., Kudo T., Katayama T. (2016). Association between Chronic Stress-Induced Structural Abnormalities in Ranvier Nodes and Reduced Oligodendrocyte Activity in Major Depression. Sci. Rep..

[142] Platt R.J., Zhou Y., Slaymaker I.M., Shetty A.S., Weisbach N.R., Kim J.A., Sharma J., Desai M., Sood S., Kempton H.R. (2017). Chd8 Mutation Leads to Autistic-like Behaviors and Impaired Striatal Circuits. Cell Rep..

[143] Lu H.-C., Tan Q., Rousseaux M.W.C., Wang W., Kim J.-Y., Richman R., Wan Y.-W., Yeh S.-Y., Patel J.M., Liu X. (2017). Disruption of the ATXN1–CIC Complex Causes a Spectrum of Neurobehavioral Phenotypes in Mice and Humans. Nat. Genet..

[144] Peñagarikano O., Lázaro M.T., Lu X.H., Gordon A., Dong H., Lam H.A., Peles E., Maidment N.T., Murphy N.P., Yang X.W. (2015). Exogenous and Evoked Oxytocin Restores Social Behavior in the Cntnap2 Mouse Model of Autism. Sci. Transl. Med..

[145] Peñagarikano O., Abrahams B.S., Herman E.I., Winden K.D., Gdalyahu A., Dong H., Sonnenblick L.I., Gruver R., Almajano J., Bragin A. (2011). Absence of CNTNAP2 Leads to Epilepsy, Neuronal Migration Abnormalities, and Core Autism-Related Deficits. Cell.

[146] Schaafsma S.M., Gagnidze K., Reyes A., Norstedt N., Månsson K., Francis K., Pfaff D.W. (2017). Sex-Specific Gene-Environment Interactions Underlying ASD-like Behaviors. Proc. Natl. Acad. Sci. USA.

[147] Boitnott A., Garcia-Forn M., Ung D.C., Niblo K., Mendonca D., Park Y., Flores M., Maxwell S., Ellegood J., Qiu L.R. (2021). Developmental and Behavioral Phenotypes in a Mouse Model of DDX3X Syndrome. Biol. Psychiatry.

[148] Ma J., Zhang L.Q., He Z.X., He X.X., Wang Y.J., Jian Y.L., Wang X., Zhang B.B., Su C., Lu J. (2019). Autism Candidate Gene DIP2A Regulates Spine Morphogenesis via Acetylation of Cortactin. PLoS Biol..

[149] Cheh M.A., Millonig J.H., Roselli L.M., Ming X., Jacobsen E., Kamdar S., Wagner G.C. (2006). En2 Knockout Mice Display Neurobehavioral and Neurochemical Alterations Relevant to Autism Spectrum Disorder. Brain Res..

[150] Moy S.S., Nadler J.J., Young N.B., Nonneman R.J., Grossman A.W., Murphy D.L., D’Ercole A.J., Crawley J.N., Magnuson T.R., Lauder J.M. (2009). Social Approach in Genetically Engineered Mouse Lines Relevant to Autism. Genes Brain Behav..

[151] Scearce-Levie K., Roberson E.D., Gerstein H., Cholfin J.A., Mandiyan V.S., Shah N.M., Rubenstein J.L.R., Mucke L. (2008). Abnormal Social Behaviors in Mice Lacking Fgf17. Genes Brain Behav..

[152] Ronesi J.A., Collins K.A., Hays S.A., Tsai N.P., Guo W., Birnbaum S.G., Hu J.H., Worley P.F., Gibson J.R., Huber K.M. (2012). Disrupted Homer Scaffolds Mediate Abnormal MGluR5 Function in a Mouse Model of Fragile X Syndrome. Nat. Neurosci..

[153] Bhattacharya A., Kaphzan H., Alvarez-Dieppa A.C., Murphy J.P., Pierre P., Klann E. (2012). Genetic Removal of P70 S6 Kinase 1 Corrects Molecular, Synaptic, and Behavioral Phenotypes in Fragile X Syndrome Mice. Neuron.

[154] Spencer C.M., Alekseyenko O., Serysheva E., Yuva-Paylor L.A., Paylor R. (2005). Altered Anxiety-Related and Social Behaviors in the Fmr1 Knockout Mouse Model of Fragile X Syndrome. Genes Brain Behav..

[155] Shu W., Cho J.Y., Jiang Y., Zhang M., Weisz D., Elder G.A., Schmeidler J., De Gasperi R., Gama Sosa M.A., Rabidou D. (2005). Altered Ultrasonic Vocalization in Mice with a Disruption in the Foxp2 Gene. Proc. Natl. Acad. Sci. USA.

[156] Enard W., Gehre S., Hammerschmidt K., Hölter S.M., Blass T., Somel M., Brückner M.K., Schreiweis C., Winter C., Sohr R. (2009). A Humanized Version of Foxp2 Affects Cortico-Basal Ganglia Circuits in Mice. Cell.

[157] Fujita E., Tanabe Y., Shiota A., Ueda M., Suwa K., Momoi M.Y., Momoi T. (2008). Ultrasonic Vocalization Impairment of Foxp2 (R552H) Knockin Mice Related to Speech-Language Disorder and Abnormality of Purkinje Cells. Proc. Natl. Acad. Sci. USA.

[158] DeLorey T.M., Sahbaie P., Hashemi E., Li W.W., Salehi A., Clark D.J. (2011). Somatosensory and Sensorimotor Consequences Associated with the Heterozygous Disruption of the Autism Candidate Gene, Gabrb3. Behav. Brain Res..

[159] Li S., Kumar P., Joshee S., Kirschstein T., Subburaju S., Khalili J.S., Kloepper J., Du C., Elkhal A., Szabó G. (2018). Endothelial Cell-Derived GABA Signaling Modulates Neuronal Migration and Postnatal Behavior. Cell Res..

[160] Orefice L.L.L., Zimmerman A.L.L., Chirila A.M.M., Sleboda S.J.J., Head J.P.P., Ginty D.D.D. (2016). Peripheral Mechanosensory Neuron Dysfunction Underlies Tactile and Behavioral Deficits in Mouse Models of ASDs. Cell.

[161] DeLorey T.M., Sahbaie P., Hashemi E., Homanics G.E., Clark J.D. (2008). Gabrb3 Gene Deficient Mice Exhibit Impaired Social and Exploratory Behaviors, Deficits in Non-Selective Attention and Hypoplasia of Cerebellar Vermal Lobules: A Potential Model of Autism Spectrum Disorder. Behav. Brain Res..

[162] Carter M.D., Shah C.R., Muller C.L., Crawley J.N., Carneiro A.M.D., Veenstra-VanderWeele J. (2011). Absence of Preference for Social Novelty and Increased Grooming in Integrin B3 Knockout Mice: Initial Studies and Future Directions. Autism Res..

[163] Wickramasekara R.N., Robertson B., Hulen J., Hallgren J., Stessman H.A.F. (2021). Differential Effects by Sex with Kmt5b Loss. Autism Res..

[164] Gemelli T., Berton O., Nelson E.D., Perrotti L.I., Jaenisch R., Monteggia L.M. (2006). Postnatal Loss of Methyl-CpG Binding Protein 2 in the Forebrain Is Sufficient to Mediate Behavioral Aspects of Rett Syndrome in Mice. Biol. Psychiatry.

[165] Guy J., Hendrich B., Holmes M., Martin J.E., Bird A. (2001). A Mouse Mecp2-Null Mutation Causes Neurological Symptoms That Mimic Rett Syndrome. Nat. Genet..

[166] Chen R.Z., Akbarian S., Tudor M., Jaenisch R. (2001). Deficiency of Methyl-CpG Binding Protein-2 in CNS Neurons Results in a Rett-like Phenotype in Mice. Nat. Genet..

[167] Nott A., Cheng J., Gao F., Lin Y.T., Gjoneska E., Ko T., Minhas P., Zamudio A.V., Meng J., Zhang F. (2016). Histone Deacetylase 3 Associates with MeCP2 to Regulate FOXO and Social Behavior. Nat. Neurosci..

[168] Martins G.J., Plachez C., Powell E.M. (2007). Loss of Embryonic MET Signaling Alters Profiles of Hippocampal Interneurons. Dev. Neurosci..

[169] Cheng Y., Wang Z.M., Tan W., Wang X., Li Y., Bai B., Li Y., Zhang S.F., Yan H.L., Chen Z.L. (2018). Partial Loss of Psychiatric Risk Gene Mir137 in Mice Causes Repetitive Behavior and Impairs Sociability and Learning via Increased Pde10a. Nat. Neurosci..

[170] Singh K., Loreth D., Pöttker B., Hefti K., Innos J., Schwald K., Hengstler H., Menzel L., Sommer C.J., Radyushkin K. (2018). Neuronal Growth and Behavioral Alterations in Mice Deficient for the Psychiatric Disease-Associated Negr1 Gene. Front. Mol. Neurosci..

[171] Runge K., Mathieu R., Bugeon S., Lafi S., Beurrier C., Sahu S., Schaller F., Loubat A., Herault L., Gaillard S. (2021). Disruption of NEUROD2 Causes a Neurodevelopmental Syndrome with Autistic Features via Cell-Autonomous Defects in Forebrain Glutamatergic Neurons. Mol. Psychiatry.

[172] Gilbert J., O’Connor M., Templet S., Moghaddam M., Di Via Ioschpe A., Sinclair A., Zhu L.Q., Xu W., Man H.Y. (2020). NEXMIF/KIDLIA Knock-out Mouse Demonstrates Autism-Like Behaviors, Memory Deficits, and Impairments in Synapse Formation and Function. J. Neurosci..

[173] Blundell J., Tabuchi K., Bolliger M.F., Blaiss C.A., Brose N., Liu X., Südhof T.C., Powell C.M. (2009). Increased Anxiety-like Behavior in Mice Lacking the Inhibitory Synapse Cell Adhesion Molecule Neuroligin 2. Genes Brain Behav..

[174] Blundell J., Blaiss C.A., Etherton M.R., Espinosa F., Tabuchi K., Walz C., Bolliger M.F., Südhof T.C., Powell C.M. (2010). Neuroligin-1 Deletion Results in Impaired Spatial Memory and Increased Repetitive Behavior. J. Neurosci..

[175] Jamain S., Radyushkin K., Hammerschmidt K., Granon S., Boretius S., Varoqueaux F., Ramanantsoa N., Gallego J., Ronnenberg A., Winter D. (2008). Reduced Social Interaction and Ultrasonic Communication in a Mouse Model of Monogenic Heritable Autism. Proc. Natl. Acad. Sci. USA.

[176] Radyushkin K., Hammerschmidt K., Boretius S., Varoqueaux F., El-Kordi A., Ronnenberg A., Winter D., Frahm J., Fischer J., Brose N. (2009). Neuroligin-3-Deficient Mice: Model of a Monogenic Heritable Form of Autism with an Olfactory Deficit. Genes Brain Behav..

[177] Pobbe R.L.H., Pearson B.L., Defensor E.B., Bolivar V.J., Young W.S., Lee H.J., Blanchard D.C., Blanchard R.J. (2012). Oxytocin Receptor Knockout Mice Display Deficits in the Expression of Autism-Related Behaviors. Horm. Behav..

[178] Sala M., Braida D., Lentini D., Busnelli M., Bulgheroni E., Capurro V., Finardi A., Donzelli A., Pattini L., Rubino T. (2011). Pharmacologic Rescue of Impaired Cognitive Flexibility, Social Deficits, Increased Aggression, and Seizure Susceptibility in Oxytocin Receptor Null Mice: A Neurobehavioral Model of Autism. Biol. Psychiatry.

[179] Macbeth A.H., Stepp J.E., Lee H.J., Young W.S., Caldwell H.K. (2010). Normal Maternal Behavior, but Increased Pup Mortality, in Conditional Oxytocin Receptor Knockout Females. Behav. Neurosci..

[180] Hayashi S., Inoue Y., Hattori S., Kaneko M., Shioi G., Miyakawa T., Takeichi M. (2017). Loss of X-Linked Protocadherin-19 Differentially Affects the Behavior of Heterozygous Female and Hemizygous Male Mice. Sci. Rep..

[181] Matsumura K., Seiriki K., Okada S., Nagase M., Ayabe S., Yamada I., Furuse T., Shibuya H., Yasuda Y., Yamamori H. (2020). Pathogenic POGZ Mutation Causes Impaired Cortical Development and Reversible Autism-like Phenotypes. Nat. Commun..

[182] Vogt D., Cho K.K.A., Lee A.T., Sohal V.S., Rubenstein J.L.R. (2015). The Parvalbumin/Somatostatin Ratio Is Increased in Pten Mutant Mice and by Human PTEN ASD Alleles. Cell Rep..

[183] Cupolillo D., Hoxha E., Faralli A., De Luca A., Rossi F., Tempia F., Carulli D. (2016). Autistic-Like Traits and Cerebellar Dysfunction in Purkinje Cell PTEN Knock-Out Mice. Neuropsychopharmacology.

[184] Cabral-Costa J.V., Andreotti D.Z., Mello N.P., Scavone C., Camandola S., Kawamoto E.M. (2018). Intermittent Fasting Uncovers and Rescues Cognitive Phenotypes in PTEN Neuronal Haploinsufficient Mice. Sci. Rep..

[185] Williams M.R., De-Spenza T., Li M., Gulledge A.T., Luikart B.W. (2015). Hyperactivity of Newborn Pten Knock-out Neurons Results from Increased Excitatory Synaptic Drive. J. Neurosci..

[186] Zhang W., Ma L., Yang M., Shao Q., Xu J., Lu Z., Zhao Z., Chen R., Chai Y., Chen J.F. (2020). Cerebral Organoid and Mouse Models Reveal a RAB39b-PI3K-MTOR Pathway-Dependent Dysregulation of Cortical Development Leading to Macrocephaly/Autism Phenotypes. Genes Dev..

[187] Nyarenchi O.M., Scherer A., Wilson S., Fulkerson D.H. (2014). Cloacal Exstrophy with Extensive Chiari II Malformation: Case Report and Review of the Literature. Child’s Nerv. Syst..

[188] Mullen B.R., Khialeeva E., Hoffman D.B., Ghiani C.A., Carpenter E.M. (2013). Decreased Reelin Expression and Organophosphate Pesticide Exposure Alters Mouse Behaviour and Brain Morphology. ASN Neuro.

[189] Coba M.P., Ramaker M.J., Ho E.V., Thompson S.L., Komiyama N.H., Grant S.G.N., Knowles J.A., Dulawa S.C. (2018). Dlgap1 Knockout Mice Exhibit Alterations of the Postsynaptic Density and Selective Reductions in Sociability. Sci. Rep..

[190] Planells-Cases R., Caprini M., Zhang J., Rockenstein E.M., Rivera R.R., Murre C., Masliah E., Montal M. (2000). Neuronal Death and Perinatal Lethality in Voltage-Gated Sodium Channel AII-Deficient Mice. Biophys. J..

[191] Tatsukawa T., Raveau M., Ogiwara I., Hattori S., Miyamoto H., Mazaki E., Itohara S., Miyakawa T., Montal M., Yamakawa K. (2019). Scn2a Haploinsufficient Mice Display a Spectrum of Phenotypes Affecting Anxiety, Sociability, Memory Flexibility and Ampakine CX516 Rescues Their Hyperactivity. Mol. Autism.

[192] Yang K., Shi Y., Du X., Wang J., Zhang Y., Shan S., Yuan Y., Wang R., Zhou C., Liu Y. (2021). SENP1 in the Retrosplenial Agranular Cortex Regulates Core Autistic-like Symptoms in Mice. Cell Rep..

[193] Deliu E., Arecco N., Morandell J., Dotter C.P., Contreras X., Girardot C., Käsper E.-L., Kozlova A., Kishi K., Chiaradia I. (2018). Haploinsufficiency of the Intellectual Disability Gene SETD5 Disturbs Developmental Gene Expression and Cognition. Nat. Neurosci..

[194] Chung C., Ha S., Kang H., Lee J., Um S.M., Yan H., Yoo Y.E., Yoo T., Jung H., Lee D. (2019). Early Correction of N-Methyl-D-Aspartate Receptor Function Improves Autistic-like Social Behaviors in Adult Shank2−/− Mice. Biol. Psychiatry.

[195] Ha S., Lee D., Cho Y.S., Chung C., Yoo Y.E., Kim J., Lee J., Kim W., Kim H., Bae Y. (2016). Cerebellar Shank2 Regulates Excitatory Synapse Density, Motor Coordination, and Specific Repetitive and Anxiety-Like Behaviors. J. Neurosci..

[196] Lim C.S., Kim H., Yu N.K., Kang S.J., Kim T.H., Ko H.G., Lee J.H., Yang J.E., Ryu H.H., Park T. (2017). Enhancing Inhibitory Synaptic Function Reverses Spatial Memory Deficits in Shank2 Mutant Mice. Neuropharmacology.

[197] Speed H.E., Kouser M., Xuan Z., Reimers J.M., Ochoa C.F., Gupta N., Liu S., Powell C.M. (2015). Autism-Associated Insertion Mutation (InsG) of Shank3 Exon 21 Causes Impaired Synaptic Transmission and Behavioral Deficits. J. Neurosci..

[198] Richter M., Murtaza N., Scharrenberg R., White S.H., Johanns O., Walker S., Yuen R.K.C., Schwanke B., Bedürftig B., Henis M. (2019). Altered TAOK2 Activity Causes Autism-Related Neurodevelopmental and Cognitive Abnormalities through RhoA Signaling. Mol. Psychiatry.

[199] Fazel Darbandi S., Robinson Schwartz S.E., Qi Q., Catta-Preta R., Pai E.L.L., Mandell J.D., Everitt A., Rubin A., Krasnoff R.A., Katzman S. (2018). Neonatal Tbr1 Dosage Controls Cortical Layer 6 Connectivity. Neuron.

[200] Huang T.N., Yen T.L., Qiu L.R., Chuang H.C., Lerch J.P., Hsueh Y.P. (2019). Haploinsufficiency of Autism Causative Gene Tbr1 Impairs Olfactory Discrimination and Neuronal Activation of the Olfactory System in Mice. Mol. Autism.

[201] Nakatani J., Tamada K., Hatanaka F., Ise S., Ohta H., Inoue K., Tomonaga S., Watanabe Y., Chung Y.J., Banerjee R. (2009). Abnormal Behavior in a Chromosome- Engineered Mouse Model for Human 15q11-13 Duplication Seen in Autism. Cell.

[202] Shemesh Y., Forkosh O., Mahn M., Anpilov S., Sztainberg Y., Manashirov S., Shlapobersky T., Elliott E., Tabouy L., Ezra G. (2016). Ucn3 and CRF-R2 in the Medial Amygdala Regulate Complex Social Dynamics. Nat. Neurosci..

[203] Johnson J.L., Stoica L., Liu Y., Zhu P.J., Bhattacharya A., Buffington S.A., Huq R., Eissa N.T., Larsson O., Porse B.T. (2019). Inhibition of Upf2-Dependent Nonsense-Mediated Decay Leads to Behavioral and Neurophysiological Abnormalities by Activating the Immune Response. Neuron.

[204] Huang L., Shum E.Y., Jones S.H., Lou C.-H., Dumdie J., Kim H., Roberts A.J., Jolly L.A., Espinoza J.L., Skarbrevik D.M. (2018). A Upf3b-Mutant Mouse Model with Behavioral and Neurogenesis Defects. Mol. Psychiatry.

[205] Ornoy A. (2009). Valproic Acid in Pregnancy: How Much Are We Endangering the Embryo and Fetus?. Reprod. Toxicol..

[206] Kolozsi E., Mackenzie R.N., Roullet F.I., Decatanzaro D., Foster J.A. (2009). Prenatal Exposure to Valproic Acid Leads to Reduced Expression of Synaptic Adhesion Molecule Neuroligin 3 in Mice. Neuroscience.

[207] Bambini-Junior V., Rodrigues L., Behr G.A., Moreira J.C.F., Riesgo R., Gottfried C. (2011). Animal Model of Autism Induced by Prenatal Exposure to Valproate: Behavioral Changes and Liver Parameters. Brain Res..

[208] Massa V., Cabrera R.M., Menegola E., Giavini E., Finnell R.H. (2005). Valproic Acid-Induced Skeletal Malformations: Associated Gene Expression Cascades. Pharmacogenet. Genom..

[209] Htway S.M., Sein M.T., Nohara K., Win-Shwe T.T. (2019). Effects of Developmental Arsenic Exposure on the Social Behavior and Related Gene Expression in C3H Adult Male Mice. Int. J. Environ. Res. Public Health.

[210] Yu C., Tai F., Song Z., Wu R., Zhang X., He F. (2011). Pubertal Exposure to Bisphenol A Disrupts Behavior in Adult C57BL/6J Mice. Environ. Toxicol. Pharmacol..

[211] Lan A., Kalimian M., Amram B., Kofman O. (2017). Prenatal Chlorpyrifos Leads to Autism-like Deficits in C57Bl6/J Mice. Environ. Health.

[212] Hanks A.N., Dlugolenski K., Hughes Z.A., Seymour P.A., Majchrzak M.J. (2013). Pharmacological Disruption of Mouse Social Approach Behavior: Relevance to Negative Symptoms of Schizophrenia. Behav. Brain Res..

[213] Gao X.M., Elmer G.I., Adams-Huet B., Tamminga C.A. (2009). Social Memory in Mice: Disruption with an NMDA Antagonist and Attenuation with Antipsychotic Drugs. Pharmacol. Biochem. Behav..

[214] Qiao H., Noda Y., Kamei H., Nagai T., Furukawa H., Miura H., Kayukawa Y., Ohta T., Nabeshima T. (2001). Clozapine, but Not Haloperidol, Reverses Social Behavior Deficit in Mice during Withdrawal from Chronic Phencyclidine Treatment. Neuroreport.

[215] Norregaard L., Gether U. (2001). The Monoamine Neurotransmitter Transporters: Structure, Conformational Changes and Molecular Gating. Curr. Opin. Drug Discov. Devel..

[216] Zhang H., Li S., Wang M., Vukusic B., Pristupa Z.B., Liu F. (2009). Regulation of Dopamine Transporter Activity by Carboxypeptidase E. Mol. Brain.

[217] Gainetdinov R.R., Caron M.G. (2001). Genetics of Childhood Disorders: XXIV. ADHD, Part 8: Hyperdopaminergic Mice as an Animal Model of ADHD. J. Am. Acad. Child Adolesc. Psychiatry.

[218] Sontag T.A., Tucha O., Walitza S., Lange K.W. (2010). Animal Models of Attention Deficit/Hyperactivity Disorder (ADHD): A Critical Review. ADHD Atten. Deficit Hyperact. Disord..

[219] Jones S.R., Gainetdinov R.R., Wightman R.M., Caron M.G. (1998). Mechanisms of Amphetamine Action Revealed in Mice Lacking the Dopamine Transporter. J. Neurosci..

[220] Gainetdinov R.R., Jones S.R., Fumagalli F., Wightman R.M., Caron M.G. (1998). Re-Evaluation of the Role of the Dopamine Transporter in Dopamine System Homeostasis1Published on the World Wide Web on 27 January 1998.1. Brain Res. Rev..

[221] Jaber M., Dumartin B., Sagné C., Haycock J.W., Roubert C., Giros B., Bloch B., Caron M.G. (1999). Differential Regulation of Tyrosine Hydroxylase in the Basal Ganglia of Mice Lacking the Dopamine Transporter. Eur. J. Neurosci..

[222] Gainetdinov R.R., Wetsel W.C., Jones S.R., Levin E.D., Jaber M., Caron M.G. (1999). Role of Serotonin in the Paradoxical Calming Effect of Psychostimulants on Hyperactivity. Science.

[223] Giros B., Jaber M., Jones S.R., Wightman R.M., Caron M.G. (1996). Hyperlocomotion and Indifference to Cocaine and Amphetamine in Mice Lacking the Dopamine Transporter. Nature.

[224] Bruno K.J., Freet C.S., Twining R.C., Egami K., Grigson P.S., Hess E.J. (2007). Abnormal Latent Inhibition and Impulsivity in Coloboma Mice, a Model of ADHD. Neurobiol. Dis..

[225] Hess E.J., Collins K.A., Wilson M.C. (1996). Mouse Model of Hyperkinesis Implicates SNAP-25 in Behavioral Regulation. J. Neurosci..

[226] Heyser C.J., Wilson M.C., Gold L.H. (1995). Coloboma Hyperactive Mutant Exhibits Delayed Neurobehavioral Developmental Milestones. Dev. Brain Res..

[227] Wilson M.C. Coloboma Mouse Mutant as an Animal Model of Hyperkinesis and Attention Deficit Hyperactivity Disorder. Proceedings of the Neuroscience and Biobehavioral Reviews.

[228] Steffensen S.C., Wilson M.C., Henriksen S.J. (1996). Coloboma Contiguous Gene Deletion EncompassingSnap Alters Hippocampal Plasticity. Synapse.

[229] Lipp H.-P., Wahlsten D. (1992). Absence of the Corpus Callosum. Genetically Defined Animal Models of Neurobehavioral Dysfunctions.

[230] Weiss R.E., Refetoff S. (2000). Resistance to Thyroid Hormone. Rev. Endocr. Metab. Disord..

[231] McDonald M.P., Wong R., Goldstein G., Weintraub B., Cheng S.Y., Crawley J.N. (1998). Hyperactivity and Learning Deficits in Transgenic Mice Bearing a Human Mutant Thyroid Hormone B1 Receptor Gene. Learn. Mem..

[232] Siesser W.B., Zhao J., Miller L.R., Cheng S.Y., McDonald M.P. (2006). Transgenic Mice Expressing a Human Mutant B1 Thyroid Receptor Are Hyperactive, Impulsive, and Inattentive. Genes Brain Behav..

[233] Thompson C.C. (2000). Thyroid Hormone Action in Neural Development. Cereb. Cortex.

[234] Nakajo S., Tsukada K., Omata K., Nakamura Y., Nakaya K. (1993). A New Brain-Specific 14-KDa Protein Is a Phosphoprotein. Its Complete Amino Acid Sequence and Evidence for Phosphorylation. Eur. J. Biochem..

[235] Totterdell S., Hanger D., Meredith G.E. (2004). The Ultrastructural Distribution of Alpha-Synuclein-like Protein in Normal Mouse Brain. Brain Res..

[236] Totterdell S., Meredith G.E. (2005). Localization of Alpha-Synuclein to Identified Fibers and Synapses in the Normal Mouse Brain. Neuroscience.

[237] Chartier-Harlin M.C., Kachergus J., Roumier C., Mouroux V., Douay X., Lincoln S., Levecque C., Larvor L., Andrieux J., Hulihan M. (2004). α-Synuclein Locus Duplication as a Cause of Familial Parkinson’s Disease. Lancet.

[238] Zarranz J.J., Alegre J., Gómez-Esteban J.C., Lezcano E., Ros R., Ampuero I., Vidal L., Hoenicka J., Rodriguez O., Atarés B. (2004). The New Mutation, E46K, of α-Synuclein Causes Parkinson and Lewy Body Dementia. Ann. Neurol..

[239] Decressac M., Mattsson B., Lundblad M., Weikop P., Björklund A. (2012). Progressive Neurodegenerative and Behavioural Changes Induced by AAV-Mediated Overexpression of α-Synuclein in Midbrain Dopamine Neurons. Neurobiol. Dis..

[240] Matsui H., Kenmochi N., Namikawa K. (2019). Age- and α-Synuclein-Dependent Degeneration of Dopamine and Noradrenaline Neurons in the Annual Killifish Nothobranchius Furzeri. Cell Rep..

[241] Senior S.L., Ninkina N., Deacon R., Bannerman D., Buchman V.L., Cragg S.J., Wade-Martins R. (2008). Increased Striatal Dopamine Release and Hyperdopaminergic-like Behaviour in Mice Lacking Both Alpha-Synuclein and Gamma-Synuclein. Eur. J. Neurosci..

[242] Faraone S.V., Doyle A.E. (2001). The Nature and Heritability of Attention-Deficit/Hyperactivity Disorder. Child Adolesc. Psychiatr. Clin. N. Am..

[243] Faraone S.V., Biederman J., Weiffenbach B., Keith T., Chu M.P., Weaver A., Spencer T.J., Wilens T.E., Frazier J., Cleves M. (1999). Brief Reports Dopamine D 4 Gene 7-Repeat Allele and Attention Deficit Hyperactivity Disorder. Am. J. Psychiatry.

[244] Grady D.L., Chi H.-C., Ding Y.-C., Smith M., Wang E., Schuck S., Flodman P., Spence M.A., Swanson J.M., Moyzis R.K. (2003). High Prevalence of Rare Dopamine Receptor D4 Alleles in Children Diagnosed with Attention-Deficit Hyperactivity Disorder. Mol. Psychiatry.

[245] Swanson J.M., Sunohara G.A., Kennedy J.L., Regino R., Fineberg E., Wigal T., Lerner M., Williams L., LaHoste G.J., Wigal S. (1998). Association of the Dopamine Receptor D4 (DRD4) Gene with a Refined Phenotype of Attention Deficit Hyperactivity Disorder (ADHD): A Family-Based Approach. Mol. Psychiatry.

[246] Avale M.E., Falzone T.L., Gelman D.M., Low M.J., Grandy D.K., Rubinstein M. (2004). The Dopamine D4 Receptor Is Essential for Hyperactivity and Impaired Behavioral Inhibition in a Mouse Model of Attention Deficit/Hyperactivity Disorder. Mol. Psychiatry.

[247] Safe S.H. (1994). Polychlorinated Biphenyls (PCBs): Environmental Impact, Biochemical and Toxic Responses, and Implications for Risk Assessment. Crit. Rev. Toxicol..

[248] Shain W., Bush B., Seegal R. (1991). Neurotoxicity of Polychlorinated Biphenyls: Structure-Activity Relationship of Individual Congeners. Toxicol. Appl. Pharmacol..

[249] Silbergeld E.K., Goldberg A.M. (1974). Lead-Induced Behavioral Dysfunction: An Animal Model of Hyperactivity. Exp. Neurol..

[250] Silbergeld E.K., Goldberg A.M. (1975). Pharmacological and Neurochemical Investigations of Lead-Induced Hyperactivity. Neuropharmacology.

[251] Zhu J., Fan F., McCarthy D.M., Zhang L., Cannon E.N., Spencer T.J., Biederman J., Bhide P.G. (2017). A Prenatal Nicotine Exposure Mouse Model of Methylphenidate Responsive ADHD-Associated Cognitive Phenotypes. Int. J. Dev. Neurosci..

[252] Buck J.M., Sanders K.N., Wageman C.R., Knopik V.S., Stitzel J.A., O’Neill H.C. (2019). Developmental Nicotine Exposure Precipitates Multigenerational Maternal Transmission of Nicotine Preference and ADHD-like Behavioral, Rhythmometric, Neuropharmacological, and Epigenetic Anomalies in Adolescent Mice. Neuropharmacology.

[253] McCarthy D.M., Morgan T.J., Lowe S.E., Williamson M.J., Spencer T.J., Biederman J., Bhide P.G. (2018). Nicotine Exposure of Male Mice Produces Behavioral Impairment in Multiple Generations of Descendants. PLoS Biol..

[254] Son G.H., Chung S., Geum D., Kang S.S., Choi W.S., Kim K., Choi S. (2007). Hyperactivity and Alteration of the Midbrain Dopaminergic System in Maternally Stressed Male Mice Offspring. Biochem. Biophys. Res. Commun..

[255] Laucht M., Esser G., Baving L., Gerhold M., Hoesch I., Ihle W., Steigleider P., Stock B., Stoehr R.M., Weindrich D. (2000). Behavioral Sequelae of Perinatal Insults and Early Family Adversity at 8 Years of Age. J. Am. Acad. Child Adolesc. Psychiatry.

[256] Mcintosh D.E., Mulkins R.S., Dean R.S. (1995). Utilization of Maternal Perinatal Risk Indicators in the Differential Diagnosis of Adhd and Uadd Children. Int. J. Neurosci..

[257] Nasevicius A., Ekker S.C. (2000). Effective Targeted Gene ‘Knockdown’ in Zebrafish. Nat. Genet..

[258] Bill B.R., Petzold A.M., Clark K.J., Schimmenti L.A., Ekker S.C. (2009). A Primer for Morpholino Use in Zebrafish. Zebrafish.

[259] Eisen J.S., Smith J.C. (2008). Controlling Morpholino Experiments: Don’t Stop Making Antisense. Development.

[260] Stainier D.Y.R., Raz E., Lawson N.D., Ekker S.C., Burdine R.D., Eisen J.S., Ingham P.W., Schulte-Merker S., Yelon D., Weinstein B.M. (2017). Guidelines for Morpholino Use in Zebrafish. PLoS Genet..

[261] Doyon Y., McCammon J.M., Miller J.C., Faraji F., Ngo C., Katibah G.E., Amora R., Hocking T.D., Zhang L., Rebar E.J. (2008). Heritable Targeted Gene Disruption in Zebrafish Using Designed Zinc-Finger Nucleases. Nat. Biotechnol..

[262] Huang P., Xiao A., Zhou M., Zhu Z., Lin S., Zhang B. (2011). Heritable Gene Targeting in Zebrafish Using Customized TALENs. Nat. Biotechnol..

[263] Wang H., Yang H., Shivalila C.S., Dawlaty M.M., Cheng A.W., Zhang F., Jaenisch R. (2013). One-Step Generation of Mice Carrying Mutations in Multiple Genes by CRISPR/Cas-Mediated Genome Engineering. Cell.

[264] Varshney G.K., Burgess S.M. (2014). Mutagenesis and Phenotyping Resources in Zebrafish for Studying Development and Human Disease. Brief. Funct. Genom..

[265] Vejnar C.E., Moreno-Mateos M.A., Cifuentes D., Bazzini A.A., Giraldez A.J. (2016). Optimized CRISPR–Cas9 System for Genome Editing in Zebrafish. Cold Spring Harb. Protoc..

[266] Hwang W.Y., Fu Y., Reyon D., Maeder M.L., Tsai S.Q., Sander J.D., Peterson R.T., Yeh J.R.J., Joung J.K. (2013). Efficient Genome Editing in Zebrafish Using a CRISPR-Cas System. Nat. Biotechnol..

[267] Moreno-Mateos M.A., Vejnar C.E., Beaudoin J.-D., Fernandez J.P., Mis E.K., Khokha M.K., Giraldez A.J. (2015). CRISPRscan: Designing Highly Efficient SgRNAs for CRISPR-Cas9 Targeting in Vivo. Nat. Methods.

[268] Liu X., Hu G., Ye J., Ye B., Shen N., Tao Y., Zhang X., Fan Y., Liu H., Zhang Z. (2020). De Novo ARID1B Mutations Cause Growth Delay Associated with Aberrant Wnt/β–Catenin Signaling. Hum. Mutat..

[269] Ishibashi M., Manning E., Shoubridge C., Krecsmarik M., Hawkins T.A., Giacomotto J., Zhao T., Mueller T., Bader P.I., Cheung S.W. (2015). Copy Number Variants in Patients with Intellectual Disability Affect the Regulation of ARX Transcription Factor Gene. Hum. Genet..

[270] Oksenberg N., Stevison L., Wall J.D., Ahituv N. (2013). Function and Regulation of AUTS2, a Gene Implicated in Autism and Human Evolution. PLoS Genet..

[271] Ramachandran K.V., Hennessey J.A., Barnett A.S., Yin X., Stadt H.A., Foster E., Shah R.A., Yazawa M., Dolmetsch R.E., Kirby M.L. (2013). Calcium Influx through L-Type Ca_V_1.2 Ca^2+^ Channels Regulates Mandibular Development. J. Clin. Investig..

[272] Patowary A., Won S.Y., Oh S.J., Nesbitt R.R., Archer M., Nickerson D., Raskind W.H., Bernier R., Lee J.E., Brkanac Z. (2019). Family-Based Exome Sequencing and Case-Control Analysis Implicate CEP41 as an ASD Gene. Transl. Psychiatry.

[273] Suls A., Jaehn J.A., Kecskés A., Weber Y., Weckhuysen S., Craiu D.C., Siekierska A., Djémié T., Afrikanova T., Gormley P. (2013). De Novo Loss-of-Function Mutations in CHD2 Cause a Fever-Sensitive Myoclonic Epileptic Encephalopathy Sharing Features with Dravet Syndrome. Am. J. Hum. Genet..

[274] Bernier R., Golzio C., Xiong B., Stessman H.A., Coe B.P., Penn O., Witherspoon K., Gerdts J., Baker C., Vulto-Van Silfhout A.T. (2014). Disruptive CHD8 Mutations Define a Subtype of Autism Early in Development. Cell.

[275] Sugathan A., Biagioli M., Golzio C., Erdin S., Blumenthal I., Manavalan P., Ragavendran A., Brand H., Lucente D., Miles J. (2014). CHD8 Regulates Neurodevelopmental Pathways Associated with Autism Spectrum Disorder in Neural Progenitors. Proc. Natl. Acad. Sci. USA.

[276] Hoffman E.J., Turner K.J., Fernandez J.M., Cifuentes D., Ghosh M., Ijaz S., Jain R.A., Kubo F., Bill B.R., Baier H. (2016). Estrogens Suppress a Behavioral Phenotype in Zebrafish Mutants of the Autism Risk Gene, CNTNAP2. Neuron.

[277] Turner T.N., Sharma K., Oh E.C., Liu Y.P., Collins R.L., Sosa M.X., Auer D.R., Brand H., Sanders S.J., Moreno-De-Luca D. (2015). Loss of δ-Catenin Function in Severe Autism. Nature.

[278] Kim O.H., Cho H.J., Han E., Hong T.I., Ariyasiri K., Choi J.H., Hwang K.S., Jeong Y.M., Yang S.Y., Yu K. (2017). Zebrafish Knockout of Down Syndrome Gene, DYRK1A, Shows Social Impairments Relevant to Autism. Mol. Autism.

[279] Sicca F., Ambrosini E., Marchese M., Sforna L., Servettini I., Valvo G., Brignone M.S., Lanciotti A., Moro F., Grottesi A. (2016). Gain-of-Function Defects of Astrocytic Kir4.1 Channels in Children with Autism Spectrum Disorders and Epilepsy. Sci. Rep..

[280] Bögershausen N., Tsai I.C., Pohl E., Kiper P.O.S., Beleggia F., Ferda Percin E., Keupp K., Matchan A., Milz E., Alanay Y. (2015). RAP1-Mediated MEK/ERK Pathway Defects in Kabuki Syndrome. J. Clin. Investig..

[281] Van Laarhoven P.M., Neitzel L.R., Quintana A.M., Geiger E.A., Zackai E.H., Clouthier D.E., Artinger K.B., Ming J.E., Shaikh T.H. (2015). Kabuki Syndrome Genes KMT2D and KDM6A: Functional Analyses Demonstrate Critical Roles in Craniofacial, Heart and Brain Development. Hum. Mol. Genet..

[282] Leong W.Y., Lim Z.H., Korzh V., Pietri T., Goh E.L.K. (2015). Methyl-CpG Binding Protein 2 (Mecp2) Regulates Sensory Function through Sema5b and Robo2. Front. Cell. Neurosci..

[283] Van Der Vaart M., Svoboda O., Weijts B.G., Espín-Palazón R., Sapp V., Pietri T., Bagnat M., Muotri A.R., Traver D. (2017). Mecp2 Regulates Tnfa during Zebrafish Embryonic Development and Acute Inflammation. DMM Dis. Model. Mech..

[284] Elsen G.E., Choi L.Y., Prince V.E., Ho R.K. (2009). The Autism Susceptibility Gene Met Regulates Zebrafish Cerebellar Development and Facial Motor Neuron Migration. Dev. Biol..

[285] Blanchet P., Bebin M., Bruet S., Cooper G.M., Thompson M.L., Duban-Bedu B., Gerard B., Piton A., Suckno S., Deshpande C. (2017). MYT1L Mutations Cause Intellectual Disability and Variable Obesity by Dysregulating Gene Expression and Development of the Neuroendocrine Hypothalamus. PLoS Genet..

[286] Miller A.C., Voelker L.H., Shah A.N., Moens C.B. (2015). Neurobeachin Is Required Postsynaptically for Electrical and Chemical Synapse Formation. Curr. Biol..

[287] Ruzzo E.K., Pérez-Cano L., Jung J.Y., Wang L.K., Kashef-Haghighi D., Hartl C., Singh C., Xu J., Hoekstra J.N., Leventhal O. (2019). Inherited and De Novo Genetic Risk for Autism Impacts Shared Networks. Cell.

[288] Ribeiro D., Nunes A.R., Gliksberg M., Anbalagan S., Levkowitz G., Oliveira R.F. (2020). Oxytocin Receptor Signalling Modulates Novelty Recognition but Not Social Preference in Zebrafish. J. Neuroendocrinol..

[289] Vecchia E.D., Di Donato V., Young A.M.J., Del Bene F., Norton W.H.J. (2019). Reelin Signaling Controls the Preference for Social Novelty in Zebrafish. Front. Behav. Neurosci..

[290] Plaster N., Sonntag C., Schilling T.F., Hammerschmidt M. (2007). REREa/Atrophin-2 Interacts with Histone Deacetylase and Fgf8 Signaling to Regulate Multiple Processes of Zebrafish Development. Dev. Dyn..

[291] Liu C.-X., Li C.-Y., Hu C.-C., Wang Y., Lin J., Jiang Y.-H., Li Q., Xu X. (2018). CRISPR/Cas9-Induced Shank3b Mutant Zebrafish Display Autism-like Behaviors. Mol. Autism.

[292] Kozol R.A., James D.M., Varela I., Sumathipala S.H., Züchner S., Dallman J.E. (2021). Restoring Shank3 in the Rostral Brainstem of Shank3ab−/− Zebrafish Autism Models Rescues Sensory Deficits. Commun. Biol..

[293] Kozol R.A., Cukier H.N., Zou B., Mayo V., De Rubeis S., Cai G., Griswold A.J., Whitehead P.L., Haines J.L., Gilbert J.R. (2015). Two Knockdown Models of the Autism Genes SYNGAP1 and SHANK3 in Zebrafish Produce Similar Behavioral Phenotypes Associated with Embryonic Disruptions of Brain Morphogenesis. Hum. Mol. Genet..

[294] Anderson J.L., Mulligan T.S., Shen M.C., Wang H., Scahill C.M., Tan F.J., Du S.J., Busch-Nentwich E.M., Farber S.A. (2017). MRNA Processing in Mutant Zebrafish Lines Generated by Chemical and CRISPR-Mediated Mutagenesis Produces Unexpected Transcripts That Escape Nonsense-Mediated Decay. PLoS Genet..

[295] Rea V., Van Raay T.J. (2020). Using Zebrafish to Model Autism Spectrum Disorder: A Comparison of ASD Risk Genes Between Zebrafish and Their Mammalian Counterparts. Front. Mol. Neurosci..

[296] Smith V., Brown N. (2014). Prenatal Valproate Exposure and Risk of Autism Spectrum Disorders and Childhood Autism. Arch. Dis. Child. Educ. Pract. Ed..

[297] Bromley R.L., Mawer G.E., Briggs M., Cheyne C., Clayton-Smith J., García-Fiñana M., Kneen R., Lucas S.B., Shallcross R., Baker G.A. (2013). The Prevalence of Neurodevelopmental Disorders in Children Prenatally Exposed to Antiepileptic Drugs. J. Neurol. Neurosurg. Psychiatry.

[298] Wood A. (2014). Prenatal Exposure to Sodium Valproate Is Associated with Increased Risk of Childhood Autism and Autistic Spectrum Disorder. Evid.-Based Nurs..

[299] Sailer L., Duclot F., Wang Z., Kabbaj M. (2019). Consequences of Prenatal Exposure to Valproic Acid in the Socially Monogamous Prairie Voles. Sci. Rep..

[300] Lee Y., Kim Y.H., Yun J.S., Lee C.J. (2013). Valproic Acid Decreases the Proliferation of Telencephalic Cells in Zebrafish Larvae. Neurotoxicol. Teratol..

[301] Baronio D., Puttonen H.A.J., Sundvik M., Semenova S., Lehtonen E., Panula P. (2018). Embryonic Exposure to Valproic Acid Affects the Histaminergic System and the Social Behaviour of Adult Zebrafish (*Danio rerio*). Br. J. Pharmacol..

[302] Zimmermann F.F., Gaspary K.V., Siebel A.M., Bonan C.D. (2016). Oxytocin Reversed MK-801-Induced Social Interaction and Aggression Deficits in Zebrafish. Behav. Brain Res..

[303] Seibt K.J., Piato A.L., da Luz Oliveira R., Capiotti K.M., Vianna M.R., Bonan C.D. (2011). Antipsychotic Drugs Reverse MK-801-Induced Cognitive and Social Interaction Deficits in Zebrafish (*Danio rerio*). Behav. Brain Res..

[304] Miller N., Greene K., Dydinski A., Gerlai R. (2013). Effects of Nicotine and Alcohol on Zebrafish (*Danio rerio*) Shoaling. Behav. Brain Res..

[305] Scerbina T., Chatterjee D., Gerlai R. (2012). Dopamine Receptor Antagonism Disrupts Social Preference in Zebrafish: A Strain Comparison Study. Amino Acids.

[306] Wang Y., Zhong H., Wang C., Gao D., Zhou Y., Zuo Z. (2016). Maternal Exposure to the Water Soluble Fraction of Crude Oil, Lead and Their Mixture Induces Autism-like Behavioral Deficits in Zebrafish (*Danio rerio*) Larvae. Ecotoxicol. Environ. Saf..

[307] Eddins D., Cerutti D., Williams P., Linney E., Levin E.D. (2010). Zebrafish Provide a Sensitive Model of Persisting Neurobehavioral Effects of Developmental Chlorpyrifos Exposure: Comparison with Nicotine and Pilocarpine Effects and Relationship to Dopamine Deficits. Neurotoxicol. Teratol..

[308] Regan S.L., Williams M.T., Vorhees C.V. (2021). Latrophilin-3 Disruption: Effects on Brain and Behavior. Neurosci. Biobehav. Rev..

[309] Chiu C.N., Rihel J., Lee D.A., Singh C., Mosser E.A., Chen S., Sapin V., Pham U., Engle J., Niles B.J. (2016). A Zebrafish Genetic Screen Identifies Neuromedin U as a Regulator of Sleep/Wake States. Neuron.

[310] Huang J., Zhong Z., Wang M., Chen X., Tan Y., Zhang S., He W., He X., Huang G., Lu H. (2015). Circadian Modulation of Dopamine Levels and Dopaminergic Neuron Development Contributes to Attention Deficiency and Hyperactive Behavior. J. Neurosci..

[311] de Calbiac H., Dabacan A., Marsan E., Tostivint H., Devienne G., Ishida S., Leguern E., Baulac S., Muresan R.C., Kabashi E. (2018). Depdc5 Knockdown Causes MTOR-Dependent Motor Hyperactivity in Zebrafish. Ann. Clin. Transl. Neurol..

[312] Yang L., Chang S., Lu Q., Zhang Y., Wu Z., Sun X., Cao Q., Qian Y., Jia T., Xu B. (2018). A New Locus Regulating MICALL2 Expression Was Identified for Association with Executive Inhibition in Children with Attention Deficit Hyperactivity Disorder. Mol. Psychiatry.

[313] Wan J., Yourshaw M., Mamsa H., Rudnik-Schöneborn S., Menezes M.P., Hong J.E., Leong D.W., Senderek J., Salman M.S., Chitayat D. (2012). Mutations in the RNA Exosome Component Gene EXOSC3 Cause Pontocerebellar Hypoplasia and Spinal Motor Neuron Degeneration. Nat. Genet..

[314] Fan Y., Yin W., Hu B., Kline A.D., Zhang V.W., Liang D., Sun Y., Wang L., Tang S., Powis Z. (2018). De Novo Mutations of CCNK Cause a Syndromic Neurodevelopmental Disorder with Distinctive Facial Dysmorphism. Am. J. Hum. Genet..

[315] O’Rawe J.A., Wu Y., Dörfel M.J., Rope A.F., Au P.Y.B., Parboosingh J.S., Moon S., Kousi M., Kosma K., Smith C.S. (2015). TAF1 Variants Are Associated with Dysmorphic Features, Intellectual Disability, and Neurological Manifestations. Am. J. Hum. Genet..

[316] Stankiewicz P., Khan T.N., Szafranski P., Slattery L., Streff H., Vetrini F., Bernstein J.A., Brown C.W., Rosenfeld J.A., Rednam S. (2017). Haploinsufficiency of the Chromatin Remodeler BPTF Causes Syndromic Developmental and Speech Delay, Postnatal Microcephaly, and Dysmorphic Features. Am. J. Hum. Genet..

[317] Pietri T., Roman A.C., Guyon N., Romano S.A., Washbourne P., Moens C.B., de Polavieja G.G., Sumbre G. (2013). The First Mecp2-Null Zebrafish Model Shows Altered Motor Behaviors. Front. Neural Circuits.

[318] Nozawa K., Lin Y., Kubodera R., Shimizu Y., Tanaka H., Ohshima T. (2017). Zebrafish Mecp2 Is Required for Proper Axonal Elongation of Motor Neurons and Synapse Formation. Dev. Neurobiol..

[319] Norton W. (2013). Towards Developmental Models of Psychiatric Disorders in Zebrafish. Front. Neural Circuits.

[320] Walitza S., Renner T.J., Dempfle A., Konrad K., Wewetzer C., Halbach A., Herpertz-Dahlmann B., Remschmidt H., Smidt J., Linder M. (2005). Transmission Disequilibrium of Polymorphic Variants in the Tryptophan Hydroxylase-2 Gene in Attention-Deficit/Hyperactivity Disorder. Mol. Psychiatry.

[321] Li J., Wang Y., Zhou R., Zhang H., Yang L., Wang B., Faraone S.V. (2007). Association between Polymorphisms in Serotonin Transporter Gene and Attention Deficit Hyperactivity Disorder in Chinese Han Subjects. Am. J. Med. Genet. Part B Neuropsychiatr. Genet..

[322] Gizer I.R., Ficks C., Waldman I.D. (2009). Candidate Gene Studies of ADHD: A Meta-Analytic Review. Hum. Genet..

[323] Ebstein R.P., Novick O., Umansky R., Priel B., Osher Y., Blaine D., Bennett E.R., Nemanov L., Katz M., Belmaker R.H. (1996). Dopamine D4 Receptor (D4DR) Exon III Polymorphism Associated with the Human Personality Trait of Novelty Seeking. Nat. Genet..

[324] Hawi Z., Dring M., Kirley A., Foley D., Kent L., Craddock N., Asherson P., Curran S., Gould A., Richards S. (2002). Serotonergic System and Attention Deficit Hyperactivity Disorder (ADHD): A Potential Susceptibility Locus at the 5-HT1B Receptor Gene in 273 Nuclear Families from a Multi-Centre Sample. Mol. Psychiatry.

[325] Nowicki M., Tran S., Chatterjee D., Gerlai R. (2015). Inhibition of Phosphorylated Tyrosine Hydroxylase Attenuates Ethanol-Induced Hyperactivity in Adult Zebrafish (*Danio rerio*). Pharmacol. Biochem. Behav..

[326] Fernandes Y., Tran S., Abraham E., Gerlai R. (2014). Embryonic Alcohol Exposure Impairs Associative Learning Performance in Adult Zebrafish. Behav. Brain Res..

[327] de Abreu M.S., Genario R., Giacomini A.C.V.V., Demin K.A., Lakstygal A.M., Amstislavskaya T.G., Fontana B.D., Parker M.O., Kalueff A.V. (2020). Zebrafish as a Model of Neurodevelopmental Disorders. Neuroscience.

[328] Carvan M.J., Loucks E., Weber D.N., Williams F.E. (2004). Ethanol Effects on the Developing Zebrafish: Neurobehavior and Skeletal Morphogenesis. Neurotoxicol. Teratol..

[329] Luchiari A.C., Salajan D.C., Gerlai R. (2015). Acute and Chronic Alcohol Administration: Effects on Performance of Zebrafish in a Latent Learning Task. Behav. Brain Res..

[330] Cleal M., Fontana B.D., Parker M.O. (2021). The Cognitive and Behavioral Effects of D-Amphetamine and Nicotine Sensitization in Adult Zebrafish. Psychopharmacology.

[331] Mech A.M., Merteroglu M., Sealy I.M., Teh M.T., White R.J., Havelange W., Brennan C.H., Busch-Nentwich E.M. (2022). Behavioral and Gene Regulatory Responses to Developmental Drug Exposures in Zebrafish. Front. Psychiatry.

[332] Lawrence T. (2001). Body Length, Activity Level, and Avoidance Learning in Zebrafish Exposed to Nicotine as Embryos. Master’s Thesis.

[333] Fann B. (2020). Effects of Nicotine on Contextual Fear Conditioning in Adult Zebrafish (*Danio rerio*). Bachelor’s Thesis.

[334] Zhang J., Peterson S.M., Weber G.J., Zhu X., Zheng W., Freeman J.L. (2011). Decreased Axonal Density and Altered Expression Profiles of Axonal Guidance Genes Underlying Lead (Pb) Neurodevelopmental Toxicity at Early Embryonic Stages in the Zebrafish. Neurotoxicol. Teratol..

[335] Lovato A.K., Creton R., Colwill R.M. (2016). Effects of Embryonic Exposure to Polychlorinated Biphenyls (PCBs) on Larval Zebrafish Behavior. Neurotoxicol. Teratol..

[336] Levin E.D., Sledge D., Roach S., Petro A., Donerly S., Linney E. (2011). Persistent Behavioral Impairment Caused by Embryonic Methylphenidate Exposure in Zebrafish. Neurotoxicol. Teratol..

[337] Spulber S., Kilian P., Wan Ibrahim W.N., Onishchenko N., Ulhaq M., Norrgren L., Negri S., Di Tuccio M., Ceccatelli S. (2014). PFOS Induces Behavioral Alterations, Including Spontaneous Hyperactivity That Is Corrected by Dexamfetamine in Zebrafish Larvae. PLoS ONE.

